# Nematicidal and insecticidal activities of halogenated indoles

**DOI:** 10.1038/s41598-019-38561-3

**Published:** 2019-02-14

**Authors:** Satish Kumar Rajasekharan, Jin-Hyung Lee, Vinothkannan Ravichandran, Jin-Cheol Kim, Jae Gyu Park, Jintae Lee

**Affiliations:** 1 School of Chemical Engineering, Yeungnam University, Gyeongsan, 38541 Republic of Korea; 2 Shandong University–Helmholtz Institute of Biotechnology, School of Life Science, Shandong University, Jinan, P. R. China; 3 Department of Agricultural Chemistry, Institute of Environmentally Friendly Agriculture, College of Agriculture and Life Sciences, Chonnam National University, Gwangju, Republic of Korea; 4 Advanced Bio Convergence Center, Pohang Technopark Foundation, Pohang, 37668 Republic of Korea

## Abstract

Parasite death via ion channel activations is the hallmark of anthelmintic and antiparasitic drugs. Glutamate gated chloride channel (GluCl) is a prominent targets for drug selection and design in parasitology. We report several iodine-fluorine based lead activators of GluCl by computational studies and structure-activity relationship analysis. 5-Fluoro-4-iodo-1H-pyrrolo [2, 3-b] pyridine and 5-iodoindole were bioactive hits that displayed *in vitro* anthelmintic and insecticidal activities against *Bursaphelenchus xylophilus*, *Meloidogyne incognita*, and *Tenebrio molitor*. Two important findings stood out: (i) 5F4IPP induced parasite death, and interacted proficiently with Gln^219^ amino acid of pentameric GluCl in docking analysis, and (ii) 5-iodoindole appeared to act by forming giant vacuoles in nematodes, which led to a form of non-apoptotic death known as methuosis. The study suggests halogenated-indoles and 1H-pyrrolo [2, 3-b] pyridine derivatives be regarded potential biocides for plant-parasitic nematodes and insects, and warrants further research on the mode of actions, and field investigations.

## Introduction

Parasitic nematodes occupy various trophic levels in food chain, and collectively disrupt the ecological balance with their peculiar feeding behaviors. They have evolved for centuries, and are notorious for causing diverse infections that are fatal to humans, animals, and plants^1^. Parasitic nematodes, especially the root-knot (*Meloidogyne* spp., Heteroderidae), and the pinewood (*Bursaphelenchus xylophilus*, Parasitaphelenchidae) nematodes are well-known plant pests^2^. *Meloidogyne* spp. cause extensive losses on commercial agricultural farms, whereas *B*. *xylophilus* causes the devastating pine wilt disease (PWD)^3,4^. PWD is a serious distress condition in several countries including China, Japan, and Korea, which results in millions of acres being framed under global quarantine^5^.

Global concerns regarding the dispersion of parasitic nematodes in ecosystems are considerable. The nematodes were uncontrollable, before the discovery of ivermectin, a broad spectrum anthelmintic and insecticide. Though, the use of ivermectin and related drugs to control parasites continues and substantially aids infection control, nematodes tend to develop drug resistance due to recurrent exposure and excessive usage. For example, the nematodes, *Trichostrongylus colubriformis* (Chromadorea, Trichostrongylidae) and *Ostertagia circumcincta* (Chromadorea, Trichostrongylidae) were recently reported to be resistant to ivermectin and related drugs^6–8^, and the spread of resistance genes by horizontal and vertical gene transfer within clades is plausible. Considering the current scenario, it is crucial to identify eco-friendly nematicides and insecticides, and such efforts are reported to be effective^9–11^.

Drugs containing an indole nucleus are remarkably used in the field pharmacology in recent years^12^. The molecular structure of indoles makes it suitable for structural modifications to develop prospective drugs with multifaceted medicinal and biological properties^13^. Many indole based drugs are currently in clinical trials and others are already approved by Food and Drug for therapeutic use^14,15^. Indole alkaloid okaramines and its derivatives have been reported to function by agonizing the invertebrate specific glutamate-gated chloride channel (GluCl)^16^.

Halo-indoles, like 5-chloroindole and 5-iodoindole, have shown to function as orthostatic receptor agonists and positive allosteric modulators^17^. Recently, studies with azaindoles have escalated, and its derivatives are supreme candidates for deriving novel therapeutic leads with significant medicinal and biological properties^18^. 7-Azaindoles consists of a pyridine fused to pyrrole ring by C-C bond, and the nucleus resembles an indole or a purine ring system thus portraying it as a bioisosteres^19^. Targeting ion channels are the hallmark of anthelmintic and antiparasitic drugs^20^. In the present study, we studied the nematicidal and insecticidal effects of several indole based iodine-fluorine compounds on model organisms, *B*. *xylophilus* (the pinewood nematode), *M*. *incognita* (the root knot nematode), and *Tenebrio molitor* (the meal worm beetle), and investigated the inhibitory activities of best hit, 5-Fluoro-4-iodo-1H-pyrrolo [2, 3-b] pyridine (5F4IPP), by various nematicidal bioassays and structure-activity relationship (SAR) studies.

## Results

### Structure-based virtual screening of indole based iodine-fluorine compounds as potent ion channel agonists

Glutamate gated chloride channel receptor (GluCl) is a potent developmental target for various nematicides and insecticides. Established macrocyclic lactone derivatives namely, abamectin, avermectin, and the ivermectin, achieve invertebrate killing by effectively binding to the GluCl and opening the ion channel, thus causing rapid chloride ion influx into cells, leading to membrane hyperpolarization^21^. Previously, we showed that indole derivatives, especially 5-iodoindole, have the ability to bind to a transmembrane site in the GluCl structure^15^. Furthermore, 679 iodine-fluorine compounds were docked with the transmembrane allosteric inter-subunit site of GluCl by molecular docking analysis with Schrodinger suite (Maestro module) and sorted based on the highest negative energy Glide docking scores. Of these compounds, several ligands had docking scores in the range of −5 kcal/moL. The docking parameters of 242 iodine-fluorine based compounds are presented in Supplementary Table 1.

**Figure 1:**
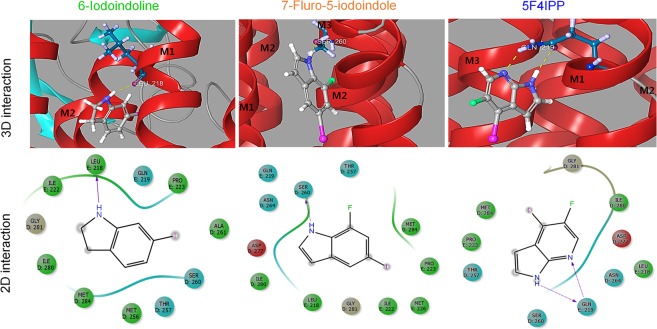
*In silico* screening of indole based iodine-fluorine compounds. Binding orientations of ligands (6-iodoindoline, 7-fluoro-5-iodoindole, and 5F4IPP) (iodine is shown in pink, fluorine in green, and backbone hydrogen bonds are shown as dotted yellow lines). The 2D image shows the interactions between ligands and surrounding amino acid residues within M2-M3 of chain D and M1-M2 of chain E (D and E in the 2D interaction diagram denote the respective chains); hydrogen bonds are shown as dotted pink arrows. Polar amino acids are shown in blue color, and the hydrophobic residues are displayed in green color.

Interestingly, the best hit in our docking simulation was 5F4IPP, which had a binding energy score of −6.05 kcal/moL (Table 1). 5F4IPP-GluCl interactions were found to be stabilized by the polar amino acid residues namely, Ser^260^, Gln ^219^, and Asn^264^ in the allosteric inter-subunit site (Fig. 1). Importantly, 5F4IPP formed two classical hydrogen bonds with the Gln^219^ of bond lengths of 1.98 and 2.19 Å.

**Table 1:** Interaction parameters of indole based iodine-fluorine aromatic compounds with GluCl in frozen state where no flexibility was given to receptor.

S. No	Compound	Structure	Purity (%)	PubChem ID	GScore (kcal/moL)	ΔG_binding_ (kcal/moL)	H- Bond (s)	Key residue (s)
1	**5-Fluoro-4-iodo-1H-pyrrolo[2**,**3-b]pyridine**		**98%**	**24229278**	**−6**.**055**	**−30**.**072**	**2**	**Gln** ^**219**^ **(2)**
2	1-(4-Iodophenyl)ethanone		98%	72869	−5.832	−28.252	1	Gln^219^
3	2-Iodobenzohydrazide		95%	615675	−5.53	−35.693	3	Ser^260^ Asn^264^ Asp^277^
4	4-iodobenzaldehyde		95%	96657	−5.506	−26.713	1	Gln^219^
5	Ethyl 6-iodo-4-oxo-chromene-2-carboxylate		95%	2775235	−5.441	−44.698	1	Thr^257^
6	4-Fluoro-2-iodo-benzoic acid		98%	12520164	−5.431	−26.412	1	Gln^219^
7	**6-Iodoindoline**		**98%**	**11615696**	**−5**.**333**	**−26**.**997**	**1**	**Leu** ^**218**^
8	2-Fluoro-6-iodo-benzoic acid		98%	2733302	−5.323	−25.305	1	Gln^219^
9	2-Hydroxy-5-iodo-benzaldehyde		98%	252612	−5.375	−31.367	1	Gln^219^
10	3-Fluoro-4-(trifluoromethoxy)aniline		98%	19436618	−5.217	−29.975	1	Ser^260^
11	Fmoc-4-iodo-L-phenylalanine		96%	2761479	−6.486	−67.489	2	Leu^218^, Gln^219^
12	**7-Fluoro-5-iodoindole**		**97%**	**66802338**	**−5**.**156**	**−25**.**475**	**1**	**Ser** ^**260**^
13	7-Fluoro-5-iodoindole-3-carboxyaldehyde		97%	97046892	−5.051	−19.412	1	Ser^260^
14	1-BOC-5-iodoindole		97%	16125958	−4.681	−31.368	1	Ser^260^
15	5-Iodindolin-2-one		98%	9838045	−4.380	−28.454	—	—
16	Iodobenzene		98%	24896121	−2.994	−20.412	—	—

Iodine and fluorine on each ring are indicated in pink and green respectively. Important compounds are indicated by bold font.

The interaction patterns between 5F4IPP, 7-fluro-5-iodoindole and 6-iodoindoline and GluCl are presented in Fig. 1. 6-Iodoindoline formed hydrogen bonds with the hydrophobic Leu^218^ residue and achieved a binding energy score of −5.33 kcal/moL (Fig. 1 and Table 1), while 7-fluoro-5-iodoindole bonded with the polar Ser^260^ residue with a binding energy score of −5.15 kcal/moL (Fig. 1 and Table 1).

It was also interesting to note that among the top thirty ligands (Supplementary Table 1), 1H-pyrrolo [2, 3-b] pyridine (7-azaindole) derivatives exhibited striking similarity in terms of receptor binding (Supplementary Fig. S1). All these hits displayed the dual hydrogen bonds with Gln^219^ exactly like those formed by 5F4IPP (Supplementary Fig. S1).

### 5F4IPP controlled the instar stages of *B*. *xylophilus*

Iodine-indole complex appears to be critical for inducing methuotic cell death in *B*. *xylophilus* and the phenotypic events observed in 5-iodoindole and 7-iodoindole treated nematodes concur with this speculation^15^. To substantiate this finding, we investigated the lethalities of 7-fluro-5-iodoindole and 1-BOC-5-iodoindole on PWN killing. Interestingly, 7-fluoro-5-iodoindole exhibited an activity similar to 5-iodoindole, but BOC-5-iodoindole failed to kill the nematodes at sub-lethal dosages, presumably due to the inhibitory effect of the tert-butyloxycarbonyl protecting group (BOC group) (Supplementary Fig. S2).

**Figure 2:**
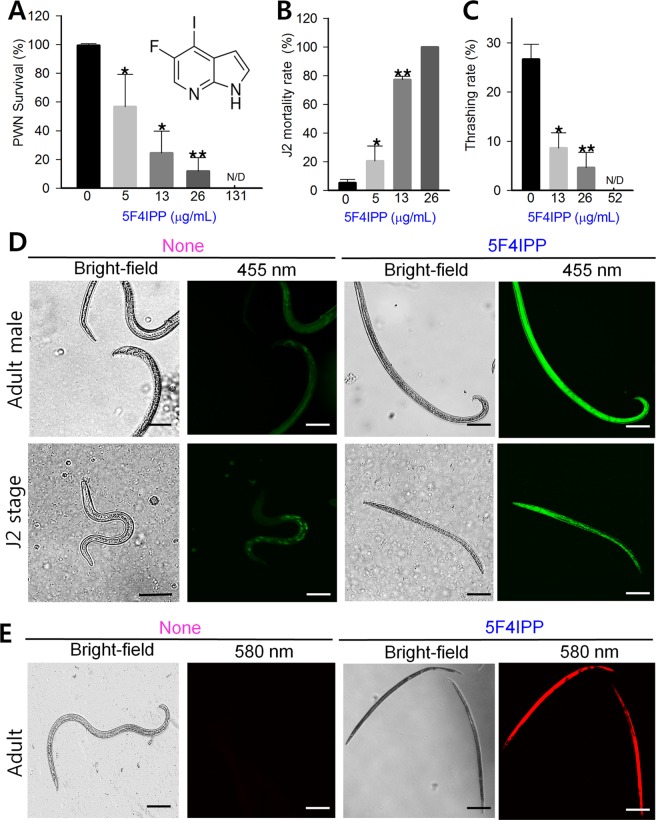
Effect of 5F4IPP on instar stages of *B*. *xylophilus*. (**A**) Survival rates of *B*. *xylophilus* following treatment with 5F4IPP, (**B**) mortality rates of *B*. *xylophilus* J2s treated with 5F4IPP, (**C**) thrashing rates of the surviving nematodes treated with 5F4IPP, (**D**) fluorescent images of live and dead nematodes (Adult male and J2) treated with or without 5F4IPP (52 µg/mL), showing discrepancy in green fluorescence emission, (**E**) fluorescent images of nematodes stained with propidium iodide after 24 h of incubation with or without 5F4IPP (52 µg/mL). Live nematodes did not uptake the propidium iodide, while the dead ones showed intense red fluorescence emission at 580 nm. The graphs show the means ± SEMs of three repetitions. *P < 0.05, **P < 0.01, and ***P < 0.001 vs. the non-treated control, Scale bars = 100 µm.

To validate the computational simulation, 5F4IPP along with other fifteen hits were tested for its *in vitro* nematicidal activities against PWN at 0.1 mM concentration. Among the tested chemicals, indole based iodine-fluorine compounds namely 5F4IPP, 7-fluoro-5-iodoindole and 6-iodoindoline revealed significant nematode killing at lower concentrations (Supplementary Fig. S2). All the three compounds exhibited a similar pattern of dose-dependent lethality. The LC_50_ and LC_90_ values of the tested compounds were calculated and are provided in Supplementary Table 2. Though at their LC_90_ values, all tested compounds exhibited similar activities against the mixed instar stages of PWN, 5F4IPP exhibited greatest efficacy at lower concentrations (LC_60_: 13.1 µg/mL = 0.05 mM) (Fig. 2A), and this was comparable to abamectin (LC_80_: 10 µg/mL) and 5-iodoindole (LC_50_ value: 12.1 µg/mL = 0.05 mM). Interestingly, nematodes treated with 5F4IPP or abamectin were devoid of any vacuoles as observed previously in 5-iodoindole treated PWN^15^.

Notably, exposure to 5F4IPP showed a significant increase in the mortality rates of *B*. *xylophilus* J2s with 100% mortality at 26 µg/mL (Fig. 2B). Representative images of live/dead adult PWN male and J2 stages are presented in Fig. 2D. The dead and live nematodes could be easily distinguished by fluorescent imaging under blue LED lights (455 nm). Dead nematodes (both adults and juveniles) showed intense green fluorescence when exited with blue light. Fluorescence levels in live control groups were relatively less (Fig. 2D). The results were concurrent with the previous findings by Coburn *et al*., though they described about blue death autofluorescence^22^. To further confirm death by 5F4IPP, the nematodes were stained with a death fluorescent marker, propidium iodide Recently it was shown to possess remarkable ability to stain dead nematodes^23^. The control groups consisting of live *B*. *xylophilus* did not take propidium iodide and were non-fluorescent, while dead nematodes in 5F4IPP treated groups showed intense red fluorescence under 580 nm emission filter (Fig. 2E).

### 5F4IPP suppressed the thrashing potential of *B*. *xylophilus*

Nematodes exhibit rapid stereospecific flexing movements and violent thrashing potentials which are regulated by the somatic motor neurons^24^. 5F4IPP significantly compromised these flexing movements. Treatments with 5F4IPP showed that the surviving populations of nematodes remained fixed to a particular position and failed to transverse across the field. The paralyzed nematodes also showed reduced or no thrashing moments as observed by time-lapse microscopic imaging (Supplementary Fig. S3). Thrashing rates were significantly lower for 5F4IPP at 13 µg/mL (9 ± 4 thrashes/min) than for controls (27 ± 3 thrashes/min) (Fig. 2C), and were reduced further by 5F4IPP at 26 µg/mL (5 ± 3 thrashes/min). Inhibition of the locomotor traits in J2s suggests its inability to disperse freely under 5F4IPP induced stress.

### 5F4IPP repressed hatching and reproductive performances in *B*. *xylophilus*

**Figure 3:**
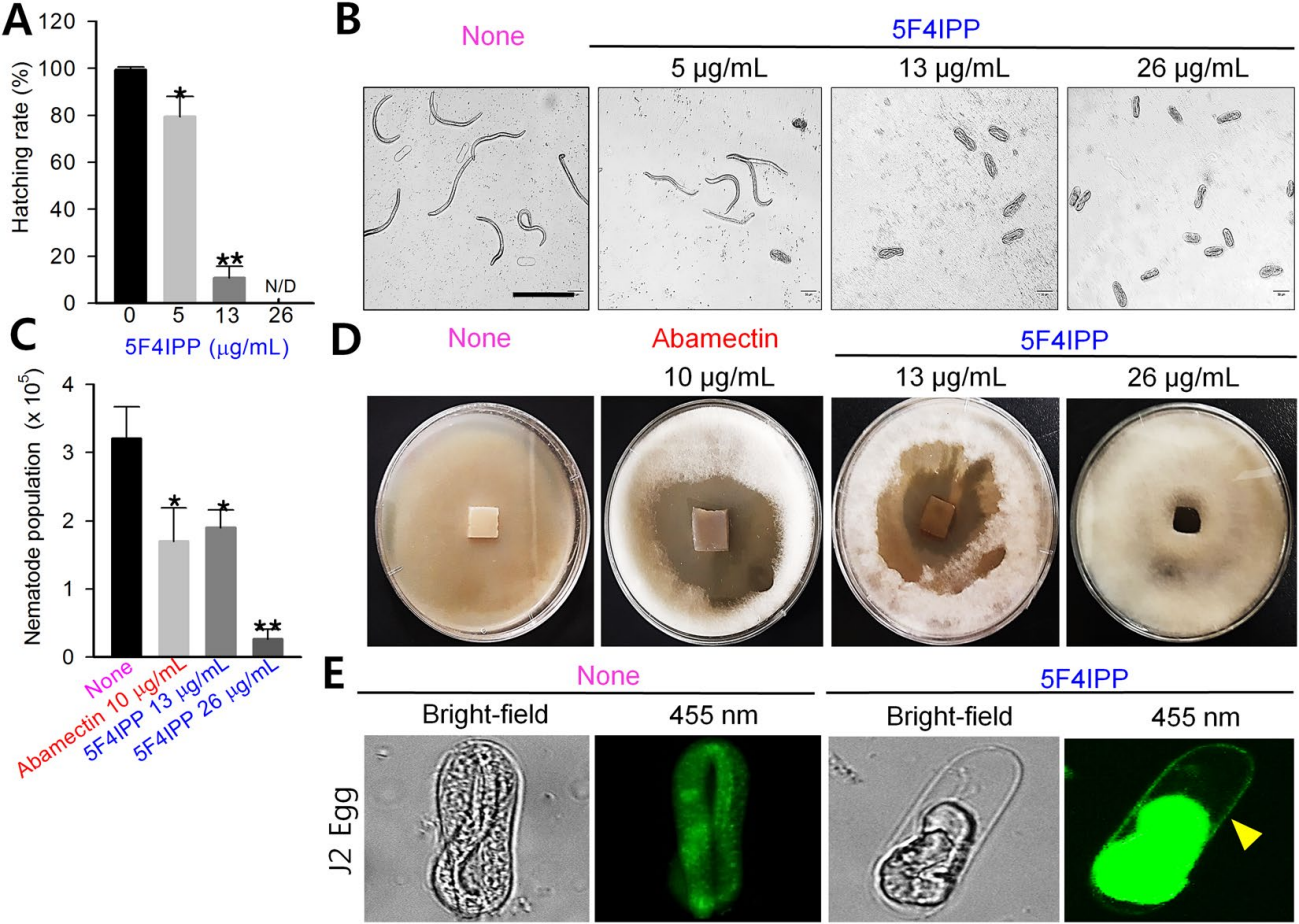
Effects of 5F4IPP on embryonic lethality, reproduction and morphology of nematode eggs. (**A**) Effects of 5F4IPP on cumulative hatching rates after 24 h, (**B**) representative images of control and 5F4IPP treated eggs, (**C**) effects of 5F4IPP and abamectin (Ab) on nematode population numbers and (**D**) areas of *B*. *cinerea* consumed by *B*. *xylophilus* after 5 d, showing differences in inhibitory activities in controls and treated groups. (**E**) Staining of J2 eggs, by fluorescent brightener 28 following treatments with 5F4IPP (131 µg/mL). The images show a live and active juvenile in control, and a shrunken and dead one in treated. The arrowhead denotes the chitin layer. The graphs show the means ± SEMs of three repetitions. *P < 0.05, **P < 0.01, and ***P < 0.001 vs. the non-treated control, Scale bars = 50 µm.

Embryogenesis is a crucial developmental factor in nematology. The hatching rate governs the proliferative potential of the nematodes. 5F4IPP significantly reduced the hatching rate of J1/J2 eggs at 13 µg/mL (Fig. 3A,B). Approximately 90% hatch inhibition was observed at 13 µg/mL which was significantly better than the standard drug abamectin (∼50%) and that of 5-iodoindole (∼75%), the other potential hit. When 5F4IPP was tested at higher concentrations (26 µg/mL), the J2 eggs failed to hatch and the J1 eggs failed to undergo the embryogenesis process suggesting that 5F4IPP arrests both early and late embryogenesis. Anticipating that a compound suppressing embryogenesis will also affect the final population numbers, we examined the abilities of 5F4IPP treated nematodes to reproduce and proliferate when fed with fungal mycelia. Furthermore, nematode counts were drastically and dose-dependently reduced. Mean nematode count for controls was 3.2 ± 0.4 × 10^5^ nematodes, while 5F4IPP treatments at 13 and 26 µg/mL resulted in counts of 1.9 ± 0.3 × 10^5^ and 0.2 ± 0.1 × 10^5^, respectively, which were comparable to those observed for abamectin (Fig. 3C). Nematodes in the control group completely consumed fungal mycelia within 5 days, whereas nematodes on plates treated with 5F4IPP failed to do so (Fig. 3D).

### 5F4IPP induced morphological abnormalities on nematodes eggs

Eggs represent the most resistant stage in the nematode life cycle, and are protected by a three layer shell comprised of chitin and cellulose^25^. Changes in the morphology of *B*. *xylophilus* eggs were observed by light and fluorescence microscopy after incubation with 5F4IPP at concentrations ranging from 13 to 131 µg/mL. In the water control, the eggs had normal shapes and embryogenesis was visualized in eggs (day 1), and J2 eggs (day 3). J2 control eggs were healthy, active and the process of hatching was observed (Supplementary Fig. S4). Treatment with 5F4IPP (26 µg/mL) significantly limited hatching rates but did not modify egg structures. However, at 131 µg/mL, several eggs were deformed and J2 eggs did not contain intact juveniles (Fig. 3E). J2 eggs treated with 5F4IPP (131 µg/mL) displayed shrunken and inactive juveniles as compared with controls, which showed live and motile juvenile. Staining of eggs with fluorescent brightener 28 (FB-28) resulted in prolific view of the chitin layer and the inner contents of the egg which fluoresced intensely, which might be not because of the stain, but due to the death fluorescence (Fig. 3E).

### 5F4IPP and 5-iodoindole effectively controlled J2s of the root-knot nematode, *Meloidogyne incognita*

**Figure 4:**
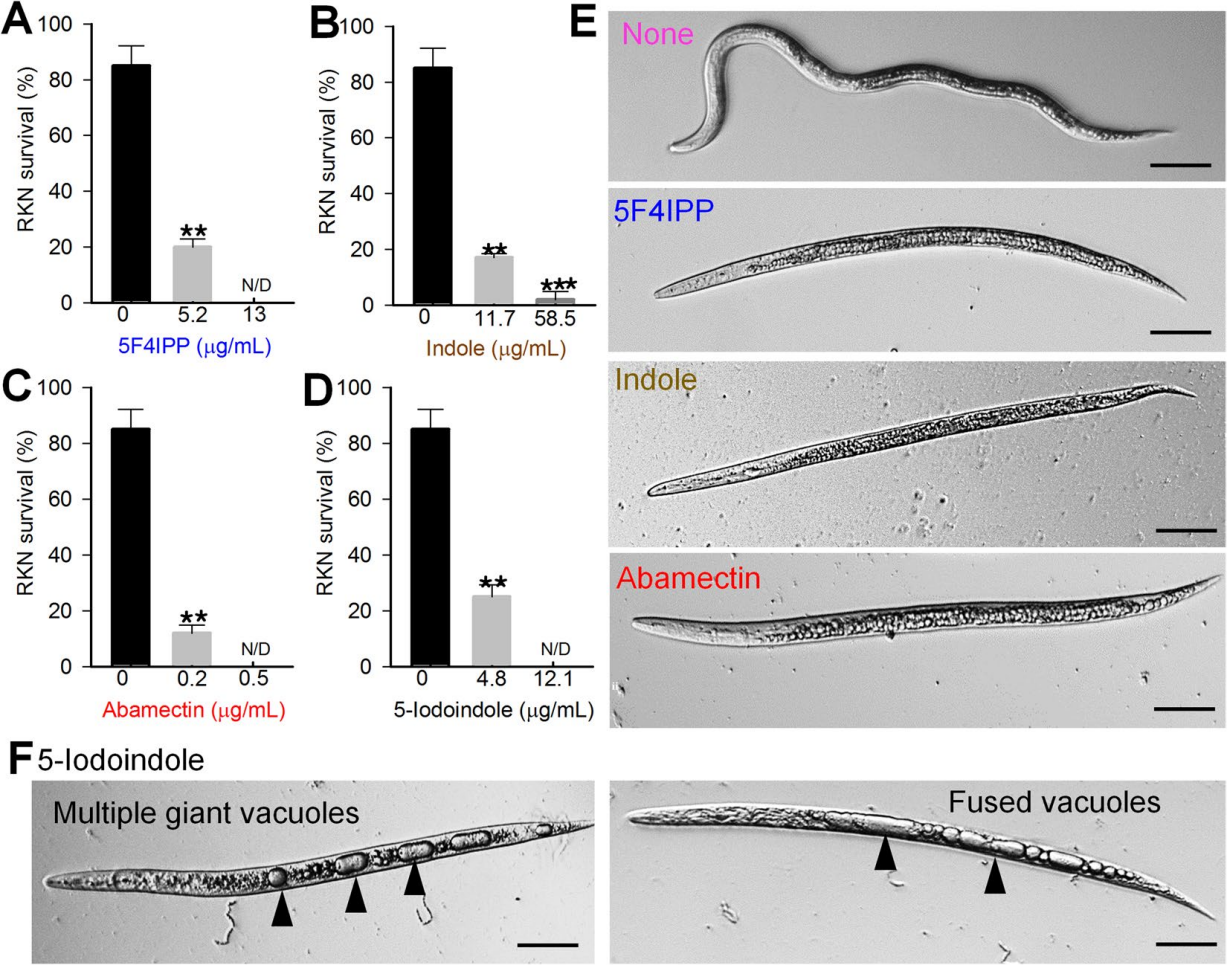
Effects of 5F4IPP and 5-iodoindole on mortality and morphology of root knot nematode, *M*. *incognita*. Effects of (**A**) 5F4IPP, (**B**) indole (**C**) abamectin, and of (**D**) 5-iodoindole on J2s of *M*. *incognita*. (**E**) Morphologies of *M*. *incognita* J2s treated with 5F4IPP (13 µg/mL), indole (58.5 µg/mL) or abamectin (0.5 µg/mL) and (**F**) morphology of *M*. *incognita* J2s treated with 5-iodoindole (12.1 µg/mL) [(i) multiple vacuoles (red arrowheads) in the body surfaces of *M*. *incognita* J2s, and (ii) giant vacuoles (yellow arrowheads) formed by vacuole fusion] suggesting methuotic death. The graphs show the means ± SEMs of two repetitions. *P < 0.05, **P < 0.01, and ***P < 0.001 vs. the non-treated control, Scale bars = 50 µm.

We next investigated the effects of indole, 5F4IPP and 5-iodoindole on J2s of the root-knot nematode, *M*. *incognita*. 5F4IPP (5.2 µg/mL = 0.02 mM) and 5-iodoindole (4 µg/mL = 0.02 mM) induced 80% J2s mortality and completely killed J2s at 12-13 µg/mL (Fig. 4A,D). 5F4IPP and 5-iodoindole had better efficacy than indole (Fig. 4B), but the nematicidal activities of both the compounds against *M*. *incognita* J2s were lower than that of abamectin (Fig. 4C), which showed complete J2 killing at concentrations as low as 0.5 µg/mL. Interestingly, at high concentration, 5-iodoindole (26 µg/mL) caused giant vacuole formation in ~50% of J2s (Fig. 4F), which were not observed in indole, abamectin, or 5F4IPP treated J2s (Fig. 4E). Furthermore, the presence of fused vacuoles in some nematodes indicated 5-iodoindole caused death by methuosis (a non-apoptotic pathway) (Fig. 4F). 5F4IPP and 5-iodoindole also achieved killing of few J3 stage *M*. *incognita* that were found along with the numerous J2s when isolated from the roots (Supplementary Fig. S5). J3 stages treated with 5-iodoindole revealed the presence of vacuoles, while few of the nematodes in 5F4IPP treated groups showed membrane shrinkage (Supplementary Fig. S5), though further investigation is warranted to clarify this occurrence.

### Methylindole derivatives did not affect *B*. *xylophilus* mortality rates

N-H group in indoles and 5F4IPP plays a significant role in binding interactions. We studied the effects of several methylindole derivatives at a fixed concentration (0.1 mM) on mortality of *B*. *xylophilus* (Supplementary Fig. S7). The tested derivatives did not kill the nematodes. Importantly, 1-methy modified derivatives, whose N-H groups were replaced with a –CH_3_ group, were non-toxic to the nematodes.

### 5F4IPP, 5-iodoindole and abamectin induced necrotic death in the instar stages of the mealworm, *Tenebrio molitor*

**Figure 5:**
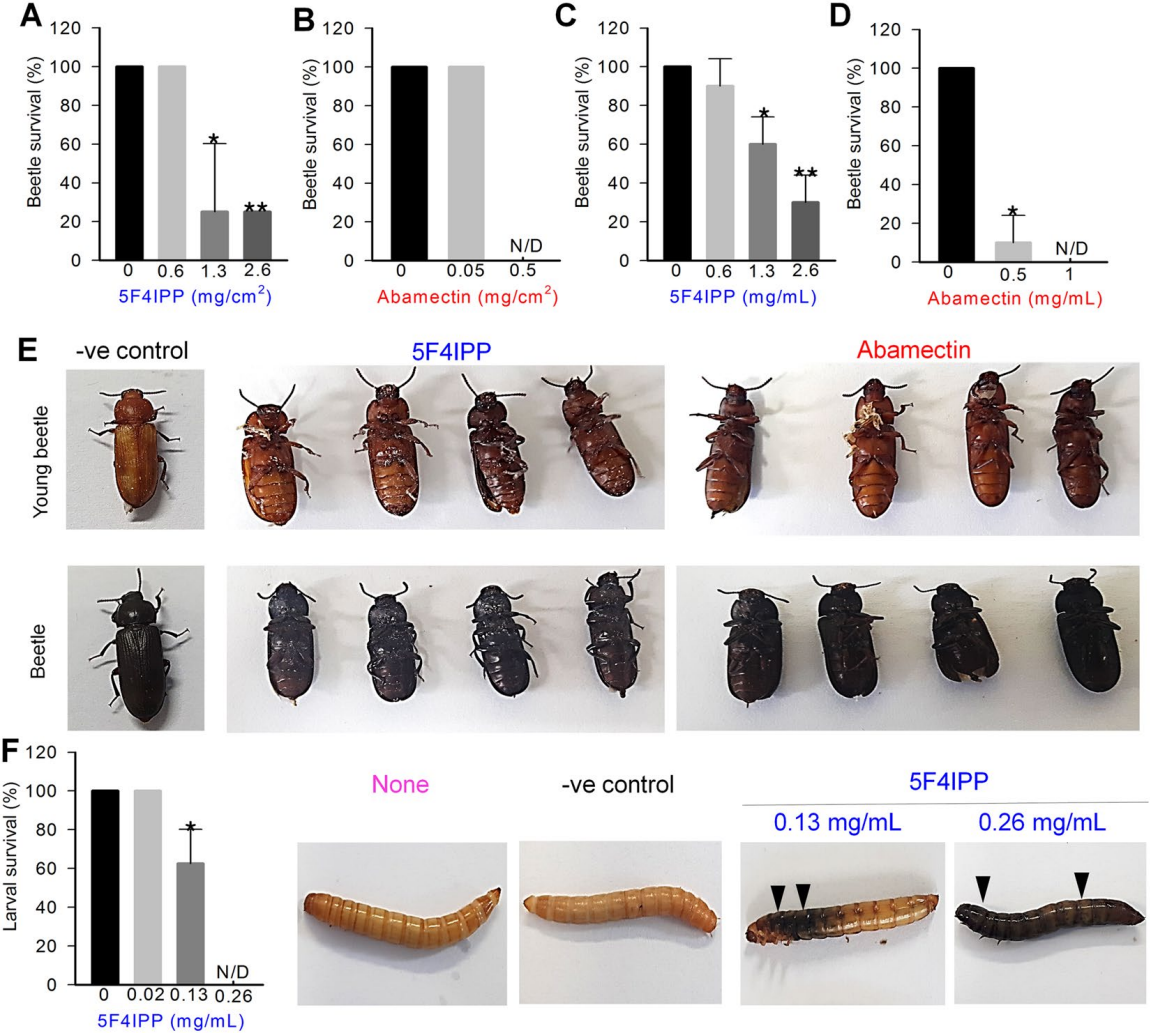
Effects of 5F4IPP and abamectin on instar stages of the model insect, *T*. *molitor*. Insecticidal activities of (**A**) 5F4IPP and (**B**) abamectin on *T*. *molitor* adult stage as determined by a surface layering insecticidal assay. Survival of *T*. *molitor* 48 h after the topical application of (**C**) 5F4IPP or (**D**) abamectin, and (**E**) toxic effects of 5F4IPP (2.6 mg/cm^2^) and abamectin (0.5 mg/cm^2^) on young (i) and adult (ii) beetles, acetone was used as a negative control. (**F**) Sequential necrotic events in the larvae and pupae of *T*. *molitor* following injection with higher concentration of 5F4IPP. 0.1% DMSO was used as a negative control. Arrowheads indicate necrotic events. The graphs show the means ± SEMs of two trials. *P < 0.05, **P < 0.01, and ***P < 0.001 vs. the non-treated control.

To investigate the effects of 5F4IPP, 5-iodoindole, and abamectin on insects, we used instar beetle larvae, pupae, and adult stage *T*. *molitor*. *T*. *molitor* is a model insect which is frequently used for screening the insecticidal activities of prospective chemicals^26^. Surface layering and topical application assays with 5F4IPP and abamectin successfully killed young (brown) and adult (black) beetles, but larval and pupal stages were resistant, and showed 100% survival rate (data not shown). The extract reason for this observation is undetermined, though we assume that larval and pupal shed their skins regularly and the skin might prevent diffusion of compounds. 5F4IPP and abamectin was successful in inducing beetle mortality when tested by surface layering assay. At 0.65 mg/cm^2^ 5F4IPP did not induce any mortality among adult beetles, though 5F4IPP layering at 1.3 or 2.6 mg/cm^2^ resulted in 80% mortality in adult and young beetles (Fig. 5A,E). The surviving beetles were motionless and near dead. The positive control abamectin efficiently killed all brown and black beetles at 0.5 mg/cm^2^ (Fig. 5B,E). 5-Iodoindole did not induce beetle mortality when tested at 1.2 or 2.4 mg/cm^2^ by surface layering method. Topical application of 2.4 mg/mL and 4.8 mg/mL of 5-iodoindole also showed not effect on beetle survival (data not shown).

However, topical application of 5F4IPP on the ventral segments of young and adult beetles produced significant mortality rates. Approximately, 70% mortality was observed when 2.6 mg/mL of 5F4IPP was applied to the pro-thorax region (Fig. 5C). Abamectin was effective even at 0.5 mg/mL and caused 100% mortality at 1 mg/mL (Fig. 5D). Intradermal injection of compounds into the larval stages of *T*. *molitor* revealed rapid necrotic events and death within 18–24 h (Fig. 5F). 5F4IPP achieved significant mortality of the larval stages of *T*. *molitor* at 0.13 mg/mL and complete killing at 0.26 mg/mL (Fig. 5F). Injection of 5F4IPP (0.26 mg/mL) also killed the pupal and beetles, though no necrotic events were noted (data not shown). On the other hand, 5-iodoindole caused 100% mortality of all the instar stages of *T*. *molitor* at concentrations as low as 0.12 mg/mL (Supplementary Fig. S6). A series of necrotic events were observed on the body surfaces of the insects with complete melanization and death at 24–48 h.

### 5F4IPP and 5-iodoindole are nontoxic to plants and animal

**Figure 6:**
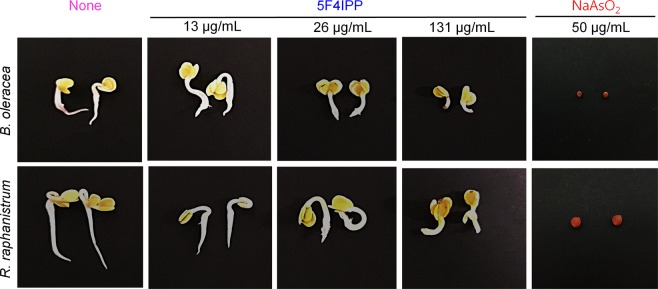
Effect of 5F4IPP on seed germination process. *B*. *oleracea* and *R*. *raphanistrum* explants grown on Murashige and Skoog agar medium supplemented with or without 5F4IPP (showing well developed root and shoot), or sodium arsenite (presenting no advancement in germination process). Germination was recorded after incubation for 2 and 4 days at 22 °C.

5F4IPP did not affect the germination of *B*. *oleracea* and *R*. *raphanistrum* seedlings in *invitro* conditions, and was relatively non-toxic to the above plants. 5F4IPP at the active concentration (13 µg/mL) did not hinder the initial sprouting or seed germination in MS agar media (Fig. 6). At higher concentrations (131 µg/mL), growth process was gradually delayed but initial germination was not significantly affected. Developments of plumules and cotyledons were not impaired, although lengths of hypocotyls were reduced at 131 µg/mL. We previously observed similar growth profiles for 5-iodoindole and abamectin^15^. The positive control, sodium arsenite (50 µg/mL), completely prevented the germination process on the agar surfaces. Sodium arsenite inhibited radicle elongation (Phase III) on Murashige and Skoog medium media. The seeds did not germinate after 4 days of incubation. The control seedlings showed normal phase III (radicle elongation) and phase IV (seedling growth) on second and fourth day respectively. Importantly, 5F4IPP was nontoxic to plants at 13 and 26 µg/mL, that is, at concentrations that killed the nematodes, which suggests its potential applications as therapeutics on pine trees and other plants. In addition, the toxicity of 5-iodoindole in a mouse model was assessed, and found it to be relatively less toxic (LD_50_ > 11040 mg/Kg by oral administration to BALB/c mice, as compared with indole (LD_50_ = ∼960 mg/Kg) (Supplementary Table 3). Notably, the LD_50_ value of 5-iodoindole reported in the present study is 138 times higher compared to oral LD_50_ of abamectin (LD_50_ = >14–80 mg/Kg in mice)^27,28^.

## Discussion

Parasitic nematodes and insects substantially hamper the sustainable management of pine trees and agricultural crops. Current study demonstrates the importance of an iodine-fluorine based pyrrolo-pyridine, 5F4IPP, as a potent compound with dual-specific activity against the plant-parasitic nematodes (*B*. *xylophilus and M*. *incognita*) and insects with a similar mechanism of action as exhibited by the macrocyclic lactone derivatives.

Pyrrolo-pyridine is structurally similar to indoles, and previously, we described the nematicidal activities of indole and 5-iodoindole against *C*. *elegans* and *B*. *xylophilus* respectively, and have suggested that the iodine-indole complex is crucial for formation of multiple vacuoles and methuotic death in pinewood nematodes^15,29^. The present study was undertaken to examine the potencies and document the antihelminthic and insecticidal effects of several other indole based iodine-fluorine compounds.

Various indole based iodine-fluorine compounds were tested for its binding interactions with GluCl of *Caenorhabditis elegans* by computational approaches. Previously, it was shown by genomic and transcriptomic studies that GluCls are widespread in protostomes which include nematodes, insects, crustaceans, flatworms, ticks and mites^21^. Moreover, *avr-14* gene that encodes an alpha-type subunit of a GluCl, is present in all parasitic clades^20^. *C*. *elegans* has served as a useful model to study the mechanism of action of ivermectins^30,31^ and hence, we used the GluCl template of *C*. *elegans* for our studies. The best sixteen were then subjected to *in vitro* evaluation against *B*. *xylophilus* (Supplementary Fig. S2) and 5F4IPP was identified as the most potent. Nematode eggs are actively protected by a three layer egg shell and embryogenesis takes place inside the shell within three days of egg laying^25,32–34^. Our study shows 5F4IPP arrests embryogenesis and is lethal to all the developmental stages of PWN.

J2s and dauers are the most resistant stages of PWN and can survive harsh environmental circumstances^35,36^. J2s of *M*. *incognita* play a vital role in inducing the root knot symptoms and collectively destroy agricultural plants^37^. We compared the efficacies of 5F4IPP and 5-iodoindole with that of abamectin and found that both compounds induced significant mortality among J2s of *B*. *xylophilus* and *M*. *incognita* (Figs 2B and 4). The process of methuosis was observed in 5-iodoindole treated *M*. *incognita* J2s as evidenced by multiple vacuoles and vacuole fusions (Fig. 4F). Several authors have reported the presence of multiple vacuoles in *M*. *incognita*^38–42^, although none have explained or emphasized the role vacuoles in inducing nematode death.

**Figure 7:**
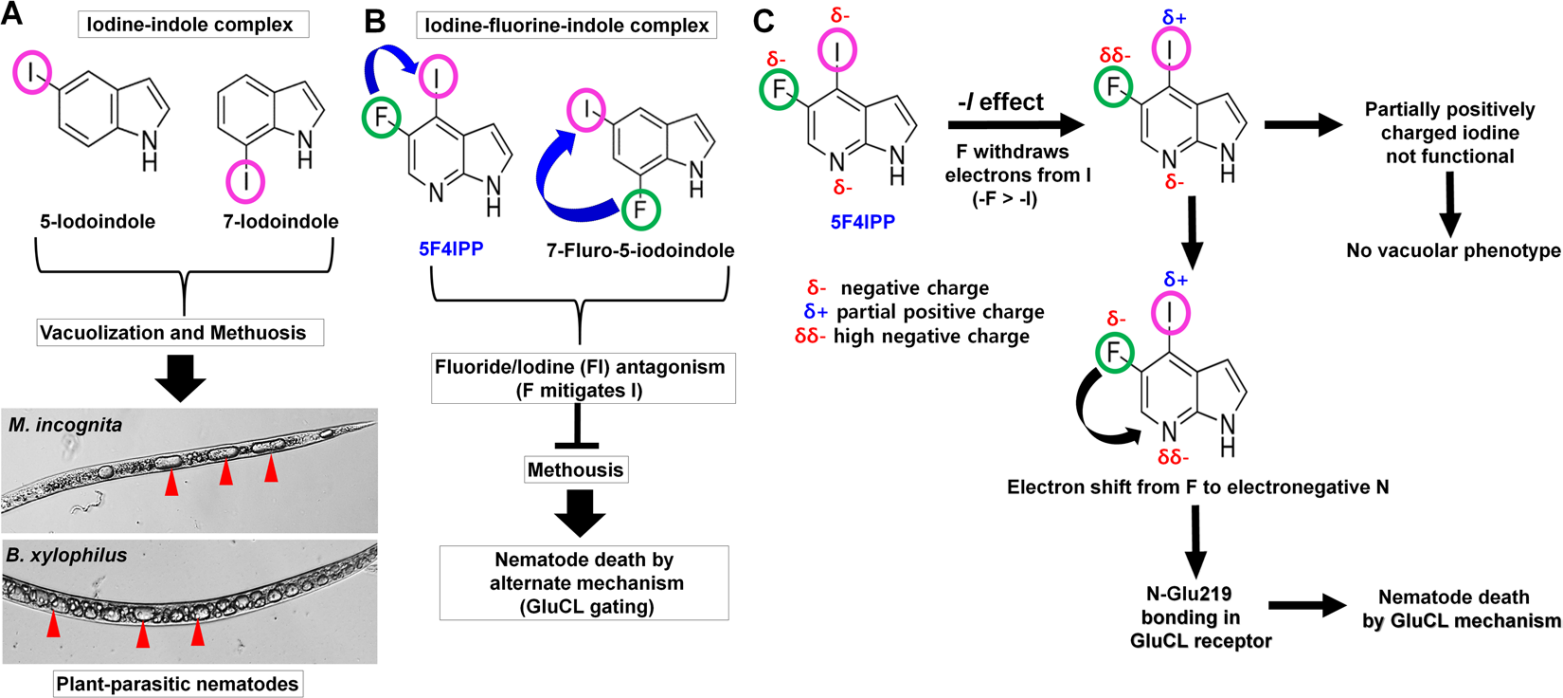
Proposed chemistry based model for induction of methousis. (**A**) Iodine-indole complex appears to play a crucial role in inducing methuosis. Iodine in 5-iodoindole and 7-iodoindole appears to induce multiple vacuoles in the body surfaces of *M*. *incognita* and *B*. *xylophilus*. Subsequent fusion to form giant vacuoles resulted in methuosis (a non-apoptotic type of cell death). (**B**) The vacuolar phenotypic was not observed in 5F4IPP or 7-fluoro-5-iodoindole treated nematodes, presumably because the fluorine acted as an iodine antagonist. Arrowheads (Red) indicate giant vacuoles, and (**C**) inductive effect induced by the electronegativity of fluorine in 5F4IPP and its biological effects.

We speculated that the death due to 5-iodoindole induced methousis in nematode is an event preceding the reversible cell injury (RCI)^15^. Here, we report the presence of multiple giant vacuoles in *M*. *incognita* (Figs 4F and 7). Taken together, our results show that at sub-lethal dosage of 5-iodoindole, several nematodes are rendered inactive due to vacuole swelling. Furthermore, we had previously shown that survived nematodes after 5-iodoindole treatment (LC_50_) can multiply when grown on fungal feed^15^.

Our study reinforces the notion that the iodine-indole complex might be essential for the induction of methuosis and that the presence of a fluorine in indole ring militates against this death pathway. The iodine in the indole ring of 5-iodoindole appears to drive this process. Importantly, no vacuolar phenotypes were observed in 5F4IPP or 7-fluoro-5-iodoindole treated nematodes, which suggests that fluorine in these indoles might mitigate methuosis by suppressing the effects of iodine (Fig. 7B). Fluorine is an iodine antagonist and completes with iodine for binding sites^43–45^. Our findings theorizes 5F4IPP activates a methuosis independent pathway to induce nematode death (Fig. 7B).

Fluorine is the most electronegative element in Group 7 elements of the periodic table with a Pauling electronegativity of 3.98 Pauling units^46^. Iodine, on the other hand is less electronegative than fluorine, and thus, when fluorine and iodine are closely associated in the indole ring, fluorine attracts electron pairs due to its electron-withdrawing inductive effect (-*I* effect) and thus, become slightly negative, while iodine would tend to lose electrons. We also propose that this electronegativity associated rationale plays an important role in mitigating the function of iodine and also interaction of ligands with the GluCl (Fig. 7C).

We also speculate that besides electronegativity, size of the halogen on indole ring also dictates the interaction pattern. From our previous findings, most of the mono-halogenated congeners like fluoroindoles, bromoindoles and chloroindoles failed to elicit methuosis nor achieved good interaction scores^15^ except for iodoindoles. Overall, ring electronegativity, size of the halogen, and subtype preference seem important for receptor interaction and methuosis^47^.

Additionally, functional importance of indole N-H hydrogen bonding with ion channels has been previously explained and demonstrated^15,48^. Indole actively forms N-H hydrogen bonds with tryptophan residues of gramicidin channel, a linear ion channel used as a model for exploring the structure and function of membrane proteins, and induces conformational and dynamic shifts within the ion channel^48,49^. Furthermore, structural modification of indole N-H by methylation (-CH_3_) abolished the function of gramicidin channel, thus confirming the importance of indole N-H-bonding. To confirm this notion in our research, we performed nematicidal assays using methyl-indole derivatives and found that 1-methyl-indole derivatives (1-methyindole-2 boric acid, and 1-methylindole-2-carboxylic acid) did not have nematicidal effects (Supplementary Fig. S7), thus confirming the importance of N-H bonds.

5F4IPP has a pyridine ring fused with pyrrole ring (Supplementary Fig. S2B), and its pyridine ring has a nitrogen atom at the para position instead of the methine group (=CH−) in benzene ring which selectively differentiates pyrroles and indoles^50,51^. 5F4IPP also has one additional electronegative nitrogen atom that can actively participate in hydrogen bonding and a highly electronegative fluorine (Fig. 7C). Gln^219^ in the M1 domain of chain D of GluCl actively forms two hydrogen bonds with 5F4IPP, one with –NH atoms of the pyrrole ring and another with the electronegative nitrogen atom of the pyridine ring. These polar interactions are further stabilized by Van der Waals interactions between the surrounding amino acids and the ligand (Fig. 1). From our docking studies, we hypotheses that 5F4IPP interacts with Gln^219^ and might be involved in some state-specific electrostatic interactions with GluCl allosteric site that potentiates the nematicidal activity. However, the crux of Gln^219^ dual H-bonding with 5F4IPP remains to be determined.

Molecular disposition of halogens, particularly iodine, on the benzene and pyrrole rings also influence the orientations of ligands in the transmembrane allosteric inter-subunit site of GluCl. As previously reported, electrophilic aromatic halogenation/substitution governs the biological activities of the aromatic compounds^52–54^. In 5F4IPP, iodine is located at position 4 and fluorine is present at position 5 of the pyridine ring, which increases proximity to Ser^260^, Gln^219^, Leu^218^, Asp^277^, and Asn^264^ of GluCl (Fig. 1).

Additionally, we tested the effects of 5F4IPP on a model insect, *T*. *molitor*. We found topical applications and surface layer insecticidal assays of 5F4IPP conducted on *T*. *molitor* beetles induced significant mortality suggesting 5F4IPP, like abamectin, has broad spectrum activity (Fig. 5). In a previous study, insecticidal assays were also performed with *Tenebrio molitor* and reported necrotic death in larva, pupa, and adults^26^. Importantly, a closely related species of *T*. *molitor*, *Alphitobius diaperinus* (the lesser mealworm, Coleoptera: Tenebrionidae), which is closely related of *Tenebrio molitor*, is a serious agricultural pest with respect to poultry-rearing and food grains storage^55^ and an intermediate host of *Hadjelia truncate* (Habronematidae), a nematode that causes pigeon death^56^. Based on our experimental observations, we believe 5F4IPP could be used to control a wide range of darkling beetles including *A*. *diaperinus*. More specifically, 5F4IPP might assist control of the pine sawyer beetle by targeting both causative agents of PWD.

Nematicides against PWN and RKN must be applied to trunks and roots, respectively, and be near nontoxic to plants. Our seed germination assays revealed 5F4IPP is relatively nontoxic to young seedlings at the tested dosage. We also show that 5-iodoindole does not have any detrimental effect on plants, and a mouse model, suggesting it as an eco-friendly agent.

## Conclusion

Abamectin have been used intensively for over thirty years to control infections and limit damage caused by nematodes and insects, but unfortunately, parasites are rapidly becoming resistant to abamectin and related drugs due to evolutionary and genetic changes. The study presents the anthelmintic and insecticidal potential of a pyrrole-pyridine compound, 5F4IPP, which cause death in plant-parasitic nematodes and insects. We deliver a chemical, biological and theoretical perceptions on the mode of action of two different halogenated compounds (5F4IPP, and 5-iodoindole), which might offer hints to elucidate the complete pathway in future. Importantly, our findings suggest iodine-indole compounds might serve as a prototypes for the developments of novel nematicides that induce methuotic death in nematodes refractory to GluCl mediated death. Overall, the study shows that the eco-friendly indole based compounds, 5F4IPP and 5-iodoindole, have potential practical applications for the eradication of plant-parasitic nematodes and diverse pests.

## Materials and Methods

### Ethics statement

The mouse experiments were approved by the Ethical Committee of Yeungnam University, Gyeongsan, Republic of Korea and the methods were carried out as per the guidelines of the Ethical Committee of Yeungnam University.

### Plant-parasitic nematodes

Pinewood nematode, *B*. *xylophilus* (supplied by Hanhong Bae and Kwang-Hyun Baek, Yeungnam University) was sub-cultured by inoculating it on a potato dextrose agar (PDA) plates containing fully grown mycelium of the neurotropic fungus, *Botrytis cinerea*. The nematodes reproduced and proliferated on the plates for a period of 7–8 days by consuming the fungi. The root knot nematode, *M*. *incognita* was maintained on tomato (*Lycopersicon esculentum* Mill. cv. Seokwang) in a greenhouse settings (28 ± 5 °C). Eggs were extracted from tomato roots infected with *M*. *incognita* using 1% sodium hypochlorite solution. Eggs were extracted from infected tomato roots using 1% sodium hypochlorite solution. The nematode eggs were collected by passage through a 45 μm sieve followed by a 25 μm sieve. Collected eggs were rinsed with distilled water and used for *in vitro* experiments and second-stage juveniles (J2s) were obtained by incubating eggs at 22 °C for up to 3 days.

### Insect model

*T*. *molitor* (Coleoptera, Tenebrionidae) larval stages were obtained from the Ye Cheon Insect Institute, Ye Cheon, Korea. Larvae were allowed to develop into pupae and adult beetles under laboratory conditions. The instar stages of *T*. *molitor* were maintained in plastic boxes (60 cm long × 40 cm wide × 12 cm deep) at 24 ± 1 °C. The stages were fed with wheat bran (12% protein, 2% lipids, 75% carbohydrates and 11% minerals/sugar) and watermelon slices. Healthy instar stages without any defects, amputations, or signs of nutritional deficiency were used in the experiments.

### Iodine-fluorine compounds and abamectin

All indole based iodine-fluorine compounds listed in Table 1 were purchased from Combi-Blocks (San Diego, CA). The commercial nematicide, abamectin, was purchased from Sigma (USA). All chemicals were dissolved and diluted in dimethyl sulfoxide (DMSO). Abamectin in DMSO served as the positive control and 0.1% DMSO as the negative control.

### *In silico* simulation assays

Molecular docking experiments were conducted as previously described^15^. Docking studies were performed to examine interactions between iodine- fluorine compounds and the crystal structure of *C*. *elegans* GluCl (a glutamate-gated chloride channel)^30,57^ retrieved from the Protein Data Bank (PDB ID: 3RHW) (http://www.rcsb.org). The receptor was prepared by Protein Preparation Wizard of Schrödinger package (Maestro v11.0). The three-dimensional structure of an inhibitory anion-selective Cys-loop receptor, the homopentameric *C*. *elegans* glutamate-gated chloride channel α (GluCl) resolved at 3.3 Å was used for docking. Detailed information about the receptor is presented in the supplementary information file. Avermectin-sensitive GluCl α receptor consisted of chains A, B, C, D, and E chains complexed with Fab and ivermectin were used. For grid generation, the receptor bound ligand (ivermectin) was removed, and the allosteric inter-subunit transmembrane site in Chain D/E interface was considered, while rest of the chains and the Fab domain were removed. Ligands were retrieved from PubChem, with PubChem ID’s provided in Supplementary Table 1, prepared by LigPrep software incorporated in Schrodinger package, and a grid was generated using a ligand diameter midpoint box with a dimensions of 18Å × 18Å × 18Å. Docking was run in a Schrodinger software 11.4 (Schrodinger Software Solutions, USA), in an extra precision (XP) flexible mode. GlideScores (GScores) and binding free energy scores (ΔG_binding_) representing the affinity of ligands with receptor were obtained from pose viewer file of docked complexes. Ligands with highest GScores for potential interactions were analyzed and those that formed significant number of hydrogen bonds as compared with the positive controls were subjected to *in vitro* assays.

### *B*. *xylophilus* mortality assay

*B*. *xylophilus* (mixed developmental stages) were transferred to a 14-ml round tube and diluted with sterile distilled water to a concentration of ~500 nematodes per 100 μL. Preliminary screening was conducted by treating nematodes with iodine-fluorine compounds at 0.1 mM. Chemically treated nematodes (100 μL) were transferred into 96-well plates (3 wells per sample) and incubated at 22 °C for 24 h. Alive and dead nematodes were then counted.

### *B*. *xylophilus* J2 mortality assay

J2s lethality was assessed as previously described with slight modification^15^. Briefly, adult nematodes were collected and transferred to microtitre plates, and 1 day later, nematodes were selectively removed by pipette and discarded. Eggs which adhered to the bottoms of plates were resuspended in sterile distilled water and allowed to hatch. J2s thus obtained was used for mortality assays. Synchronized J2s (∼100 nematodes) were treated with 5F4IPP (0, 13, or 26 µg/mL) or abamectin (10 µg/mL) and incubated at 22 °C for 24 h. Experiments were conducted in three repetitions and images were acquired using an iRiS™ Digital Cell Imaging System.

The mortality rates (M) of L2s were calculated using Abbott’s formula^58^.

$$M=[({\rm{Mt}}-{\rm{Mc}})/(100-{\rm{Mc}})]\times 100.$$

where Mt and Mc represent mortality percentages for treated and non-treated controls.

### *B*. *xylophilus* population inhibition assay

Instar stages of *B*. *xylophilus* (∼500) were initially treated with 5F4IPP (0, 13 and 26 µg/mL) or abamectin (10 µg/mL) and incubated for 24 h at 22 °C as described by Cheng *et al*., 2016^4^. After 24 h, approximately, 100 nematodes were randomly picked, counted and placed at the center of *B*. *cinerea* plates and allowed to grow and reproduce for 7 d. When the *B*. *cinerea* has been completely consumed by nematodes in control plates, nematodes were extracted from plates using sterilized distilled water. Nematodes were serially diluted using sterilized distilled water and numbers of nematodes in 100 µL suspensions were counted using an iRiS™ Digital Cell Imaging System (Logos Bio Systems, Korea). The reproduction rates (P_f_/P_i_) (where P_f_ = final nematode population and P_i_ = initial nematode population) of nematodes were calculated.

### *B*. *xylophilus* thrashing assay

*B*. *xylophilus* movements in liquid culture were characterized by stereotypical flexing around body midpoints^4^. Thrashing movements of synchronized juveniles (J2s) were monitored by placing them in sterilized distilled water. Numbers of thrashes were counted manually for 1 minute; imaging was performed using an iRiS™ Digital Cell Imaging System at a magnification of 10X. A thrashing movement was defined as movement of the head in one direction then the other repetitively. Nematodes that displayed unusual twisting or coiling movements or remain motionless were not considered.

### Hatch inhibition assay of PWN

Egg hatching experiments were conducted using established procedures^38^. To estimate hatching rates (HRs), adult nematodes were collected and transferred to microtitre plates and removed by pipette 1 day later and discarded. Eggs that adhered to plate bottoms were resuspended in sterile distilled water, collected, suspended in sterilized distilled water and treated with 5F4IPP. These suspensions (100 µL; ~100 eggs) were transferred to the wells of a 24-well tissue culture plate and incubated at 22 °C. Patent J2s (determined by stimulation with a fine wire) and eggs were counted after incubation for 24 h. Six repetitions of each treatment were performed and the experiment was repeated twice. Hatch rate (HR) percentages were calculated as follows:

$${\rm{HR}}( \% )=[{\rm{juveniles}}/({\rm{eggs}}+{\rm{juveniles}})\times 100].$$

### *M*. *incognita* J2 mortality assay

J2s and eggs were isolated as previously described with slight modification^38^. *M*. *incognita* J2s (~50 nematodes) were treated with abamectin, indole, 5-iodoindole or 5F4IPP separately and incubated at 22 °C for 24 h. The experiments were conducted in two repetitions and values were expressed a mean ± SEM. Percentage nematode survival was calculated by counting numbers of living and dead nematodes in control and experimental groups.

### Surface layer insecticidal assays

*T*. *molitor* (Coleoptera, Tenebrionidae) was used as a model organism for all the insecticidal assays^26^. For conducting surface layer insecticidal assays, each concentration (100 µL) of compounds (Abamectin, 5F4IPP, and 5-iodoindole dissolved in acetone were layered on to the surface of glass coverslip (2 cm^2^), with a micropipette, and then spread uniformly. After evaporation of acetone at room temperature, healthy larvae and adult beetle of same size (~200 mg) were introduced without food into 6-well microtitre plate and covered with a lid. Mortality, based on melanization and response to touch with a sterile forcep, was determined by counting 48 h later. Glass coverslips treated with acetone were used as controls.

### Topical application assay

Topical application assays were conducted using in acetone as solvent as previously described^59^. Each concentration (2–5 µL) of compounds were topically applied to the dorsal side, on the thorax region of young and adult beetles of similar size (approximately 200 mg) using a micropipette. After evaporation of acetone at room temperature, the plates were covered with a lid and incubated at 24 °C. The mortality of the insects were counted after 24, 48 and 72 h of incubation. Mortalities, based on melanization and response to touch with a sterile forcep, were counted after 24, 48 and 72 h of incubation.

### Injection assay

Injection of the test compounds was performed to understand the rapid effect of compounds on cells by established procedures^60^. 10 µL of 5-iodoindole 5F4IPP and abamectin in 0.1–1% DMSO were injected into the hemocoel, second visible sternite, in the ventral side of *T*. *molitor* larva, pupa and adults weighing approximately 190–200 mg. Injections were performed using a sterile ultra-fine short needles (0.3 mL volume).0.1% DMSO was used as the negative control. The mortality of the insects were counted after 24, 48 and 72 h of incubation. Insect mortalities were scored based on melanization and response to touch with a sterile forcep after 24, 48 and 72 h of incubation.

### Plant germination assay

The toxic effect of 5F4IPP on seeds were assessed by conducting germination experiments on Murashige and Skoog agar plates^61–63^. *B*. *oleracea* and *R*. *raphanistrum* seeds were initially soaked in sterile distilled water for a day, washed with 1 mL of 100% ethanol, sterilized with 1 mL of 50% commercial bleach (3% sodium hypochlorite) for 15 min, and rinsed 5 times with 1 mL of sterilized water. The seeds were then pressed into agar germination plates containing 0.86 g/L (0.2×) Murashige and Skoog medium and 0.7% bacto-agar with or without 5F4IPP. Plates were then incubated at 22 °C and images were captured after incubation for 4 days. Sodium arsenite (NaAsO_2_) (Sigma Aldrich, USA) was used as a positive control for toxicity testing.

### Phase-contrast and fluorescence imaging

Phase-contrast and the fluorescence images of nematodes and eggs were acquired using a fluorescent microscope (iRiS™ Digital Cell Imaging System (Logos Bio Systems, Korea). Propidium iodide (Sigma Aldrich, USA) was used to stain nematodes treated with 5F4IPP. After 24 hours of incubation with the chemical, propidium iodide was added to the respective wells at a final concentration of 20 µM, and the plates were incubated at 25 °C for 15 minutes. The nematodes were de-stained in distilled water and imaged under a fluorescent microscope by exiting it with a green LED light. For egg fluorescence staining, a solution containing a mixture of J2 nematode eggs previously treated with or without 5F4IPP were stained with 0.1 mg/mL of fluorescence brightener 28 (Sigma, USA) and incubated for 10 min. The eggs were washed twice with distilled water and observed under the microscope equipped with a blue LED lights.

### Mice toxicity assay

All the experiments performed were in accordance with the ethical regulations in animal research, and was approved by IACUC (Institutional Animal Care and Use Committee) of Pohang Technopark, Korea (Approval code: ABCC-201708). 40 male mice (Balb/c, 6weeks, 20–23 g) supplied by Orient-bio Ltd, Korea were housed in an intensive laboratory animal care unit for a week at 22 ± 3 °C, with timely supplement of food pellets and drinking water. A twelve-hour day and night cycle was maintained in the laboratory animal care unit. The lead compounds, 5-iodoindole and indole, were tested for their toxic effects on BALB/c mice by oral administration via sonde gavage. The chemicals were solubilized in DMSO, which was also used as a vehicle control. All efforts were taken to minimize suffering and mice were sacrificed by carbon dioxide to release pain. The treatments were supervised for 24 h and the mortality of mice were recorded.

### Statistical analysis

All the experiments on nematodes were performed in two trials with six repetitions and values are expressed as means ± SEMs. Statistical significance was determined by pair-wise testing using the Students’ *t*-test, and was accepted for *p* values of <0.05, 0.01, or <0.0001 as indicated.

## Supplementary information

Supplementary Information

## Data Availability

All data generated or analyzed during this study are included in this published article (and its Supplementary Information files).
