# Supplementary material for: Nematicidal and insecticidal activities of halogenated indoles
Source: Sci Rep. 2019 Feb 14;9:2010. doi: 10.1038/s41598-019-38561-3 (PMC6375993; doi:10.1038/s41598-019-38561-3)
Supplement: Supplementary file 1 — Supplementary Information [file 41598_2019_38561_MOESM1_ESM.docx]

**Supporting Information**

**Nematicidal and insecticidal activities of halogenated indoles**

Satish Kumar Rajasekharan^a^, Jin-Hyung Lee^a^, Vinothkannan Ravichandran^b^, Jin-Cheol Kim^c,^ Jae Gyu Park^d^, and Jintae Lee^a^*

^a^School of Chemical Engineering, Yeungnam University, Gyeongsan, 38541, Republic of Korea

^b^Shandong University–Helmholtz Institute of Biotechnology, School of Life Science, Shandong University, Jinan, P. R. China.

^c^Department of Agricultural Chemistry, Institute of Environmentally Friendly Agriculture, College of Agriculture and Life Sciences, Chonnam National University, Gwangju, Republic of Korea.

^d^Advanced Bio Convergence Center, Pohang Technopark Foundation, Pohang 37668, Republic of Korea.

**Supplementary methods**

***In silico* studies.**

*C. elegans* glutamate-gated chloride channel (GluCl) in complex with Fab and ivermectin was used for docking analysis. Detailed information about the receptor, and the grid is presented below,

1. Entity ID 1 (Avermectin-sensitive glutamate-gated chloride channel GluCl alpha) consisting for chain A, B, C, D, and E were used. The number of amino acids in one entire subunit was 347 amino acid.

2. Entity ID 2 and 3 (Mouse monoclonal Fab fragment, heavy/light chain) consisting of chains F, G, H, I, J and K, L, M, N, O were removed during initial processing step.

3. Further, Chains A, B, and C were also removed and only Chain D and E were used for further analysis.

4. The ivermectin bound transmembrane domain of Chain D/E interface were selected for grid generation. The bound ivermectin was removed and grid was generated for computational studies.

**Supplementary Table 1.** Docking parameters of 242 iodine-fluorine aromatic and aliphatic compounds with the active site of GluCl.

| **S. No** | **Pubchem ID** | **IUPAC Name** | **Glide Gscore (Kcal/moL)** | **ΔG_binding_**  **(Kcal/moL)** | **No of H-bonds** | **Bond forming residues** |
| --- | --- | --- | --- | --- | --- | --- |
|  | 61479 | (2S)-2-(9H-Fluoren-9-ylmethoxycarbonylamino)-3-(4-Iodophenyl)propanoic acid | -6.486 | -67.489 | 2 | Leu^218^, Gln^219^ |
|  | 46737870 | N-(5-Iodo-2-oxo-1H-pyridin-3-yl)acetamide | -6.298 | -42.546 | 1 | Leu^218^, |
|  | 24229278 | 5-Fluoro-4-iodo-1H-pyrrolo[2,3-b]pyridine | -6.055 | -30.072 | 2 | Gln^219^ |
|  | 49761630 | 5-Fluoro-3,4-diiodo-1055H-pyrrolo[2,3-b]pyridine | -5.918 | -33.295 | 2 | Gln^219^ |
|  | 72869 | 1-(4-Iodophenyl)ethenone | -5.832 | -28.252 | 1 | Gln^219^ |
|  | 46738010 | Tert-butyl N-[(4-iodophenyl)methyl]carbamate | -5.717 | -42.978 | 1 | Leu^218^, |
|  | 15037679 | 3-(4-Iodophenyl)benzoic acid | -5.617 | -35.697 | 1 | Gln^219^ |
|  | 615675 | 2-Iodobenzohydrazide | -5.53 | -35.693 | 3 | Ser^260^,  Asn^264^,  Asp^277^, |
|  | 50989276 | 4-Iodo-2,3-dimethoxy-5-(trifluoromethyl)pyridine | -5.526 | -30.158 | 1 | Gln^219^ |
|  | 96657 | 4-Iodobenzaldehyde | -5.506 | -26.713 | 1 | Gln^219^ |
|  | 2775235 | Ethyl 6-iodo-4-oxo-chromene-2-carboxylate | -5.441 | -44.698 | 1 | Thr^267^ |
|  | 12520164 | 4-Fluoro-2-iodo-benzoic acid | -5.431 | -26.412 | 1 | Gln^219^ |
|  | 57345822 | 7-Iodo-4H-1,4-benzoxazin-3-one | -5.378 | -36.099 | 2 | Gln^219^  Asn^264^ |
|  | 85111 | 2-Quinolylhydrazine | -5.461 | -28.42 | 2 | Ser^260^, Asp^277^ |
|  | 11615696 | 6-Iodoindoline | -5.333 | -26.997 | 1 | Leu^218^, |
|  | 49761643 | 5-Fluoro-6-iodo-1H-pyrrolo[2,3-b]pyridine-4-carboxylic acid | -5.33 | -35.87 | 2 | Gln^219^ |
|  | 2733302 | 2-Fluoro-6-iodo-benzoic acid | -5.323 | -25.305 | 1 | Gln^219^ |
|  | 14581385 | 6-Fluoronaphthalene-1-carboxylic acid | -5.307 | -29.611 | 1 | Gln^219^ |
|  | 29012 | (4-Iodophenyl)methanol | -5.3 | -27.734 | 1 | Gln^219^ |
|  | 22320308 | 3-Fluoro-2-iodo-benzoic acid | -5.295 | -26.603 | 1 | Gln^219^ |
|  | 56777166 | Methyl (2S)-2-amino-3-(4-hydroxy-3,5-diiodo-Phenyl)propanoate;hydrochloride | -5.707 | -38.687 | 1 | Gln219 |
|  | 50989425 | 5-Fluoro-6-iodo-1H-pyrrolo[2,3-b]pyridine-4-carbaldehyde | -5.285 | -37.738 | 2 | Gln^219^ |
|  | 16495276 | Ethyl 3-amino-3-(4-iodophenyl)propanoate;hydrochloride | -5.311 | -33.918 | 1 | Thr^265^ |
|  | 53216611 | 3-(2-Iodophenyl)benzoic acid | -5.268 | -38.632 | 1 | Ser^260^ |
|  | 24229225 | 5-Fluoro-3-iodo-1H-pyrrolo[2,3-b]pyridine | -5.251 | -32.528 | 2 | Gln^219^ |
|  | 252612 | 2-Hydroxy-5-iodo-benzaldehyde | -5.375 | -31.367 | 1 | Gln^219^ |
|  | 10890644 | 5-Iodo-1,7-dihydropyrrolo[2,3-d]pyrimidin-4-one | -5.244 | -33.351 | 2 | Gln^219^ |
|  | 74885 | 1,1,1,2,2,3,3,4,4,5,5,6,6,7,7,8,8-Heptadecafluoro-10-iodo-decane | -5.228 | -29.341 | - | - |
|  | 74891924 | 4,5-Diiodo-1-tetrahydropyran-2-yl-pyrazole | -5.224 | -34.312 | 1 | Gln^219^ |
|  | 19436618 | 3-Fluoro-4-(trifluoromethoxy)aniline | -5.217 | -29.975 | 1 | Ser^260^ |
|  | 74891924 | 4,5-Diiodo-1-tetrahydropyran-2-yl-pyrazole | -5.211 | -36.784 | 1 | Gln^219^ |
|  | 45480481 | Ethyl 5-iodo-1H-pyrazole-4-carboxylate | -5.189 | -34.259 | 2 | Gln^219^ |
|  | 46318064 | 8-Iodo-3,4-dihydro-2H-pyrano[3,2-c]pyridine | -5.175 | -29.051 | 1 | Gln^219^ |
|  | 12060 | 3-Iodobenzoic acid | -5.114 | -26.461 | 1 | Gln^219^ |
|  | 44754754 | 2-Fluoro-4-iodo-pyridine-3-carboxylic acid | -5.108 | -27.618 | 1 | Gln^219^ |
|  | 49761633 | 5-Fluoro-6-iodo-1H-pyrrolo[2,3-b]pyridine | -5.072 | -33.706 | 2 | Gln^219^ |
|  | 42300 | (3-Iodophenyl)methanol | -5.068 | -28.259 | 2 | Gln^219^  Ser^260^ |
|  | 3681971 | Ethyl 6-iodoimidazo[1,2-a]pyridine-2-carboxylate | -5.36 | -43.395 | 1 | Thr^257^ |
|  | 17998789 | 1-(6-Iodo-3,4-dihydro-2H-1,8-naphthyridin-1-yl)-2,2-Dimethyl-propan-1-one | -5.052 | -35.949 | - | - |
|  | 46318103 | 2-Fluoro-4-iodo-6-pyrrolidin-1-yl-pyridine | -5.027 | -31.915 | - | - |
|  | 16495276 | Ethyl 3-amino-3-(4-iodophenyl)propanoate;hydrochloride | -5.052 | -34.409 | 1 | Thr^285^ |
|  | 107629 | (2-Iodophenyl)methanol | -5.005 | -27.855 | 1 | Gln^219^ |
|  | 29934881 | 1-(5-Fluoro-2-iodo-phenyl)ethanone | -5.003 | -32.436 | 1 | Thr^257^ |
|  | 16217058 | 2-Fluoro-5-iodo-benzaldehyde | -5.002 | -28.553 | 1 | Gln^219^ |
|  | 12085 | 4-Iodobenzoic acid | -4.99 | -26.183 | 1 | Gln^219^ |
|  | 77529 | 2-Iodobenzamide | -4.98 | -30.019 | 2 | Gln^219^ |
|  | 53216610 | 3-(3-Iodophenyl)benzoic acid | -4.96 | -35.959 | 1 | Thr^257^ |
|  | 15209920 | 2-Hydroxy-3-iodo-benzaldehyde | -5.197 | -23.389 | 1 | Gln^219^ |
|  | 6941 | 2-Iodobenzoic acid | -4.947 | -23.953 | 2 | Gln^219^  Asp^277^, |
|  | 143542 | Ethyl 3-iodobenzoate | -4.941 | -31.605 | 1 | Gln^219^ |
|  | 49761553 | 8-Iodo-2,3-dihydro-[1,4]dioxino[2,3-b]pyridine | -4.914 | -29.937 | 1 | Gln^219^ |
|  | 46315209 | 3-Iodo-5-(trifluoromethyl)-1H-pyridin-2-one | -4.936 | -32.898 | 1 | Gln^219^ |
|  | 736854 | 3-Hydroxy-4-iodo-benzoic acid | -4.918 | -28.446 | 1 | Thr^257^ |
|  | 49761554 | 7-Iodo-2,3-dihydro-[1,4]dioxino[2,3-b]pyridine | -4.897 | -26.504 | 1 | Gln^219^ |
|  | 50989363 | 2,3-Diiodo-5-(trifluoromethyl)pyridine | -4.892 | -28.142 | 1 | Gln^219^ |
|  | 15209920 | 2-Hydroxy-3-iodo-benzaldehyde | -5.514 | -28.365 | 1 | Gln^219^ |
|  | 50989440 | (5-Fluoro-3-iodo-2-pyridyl) trifluoromethanesulfonate | -4.858 | -34.647 | 1 | Ser^260^ |
|  | 50986523 | (5-Fluoro-6-iodo-1-triisopropylsilyl-pyrrolo[2,3-b]pyridin-4-yl)-trimethyl-silane | -4.843 | -48.654 | - | - |
|  | 56777166 | Methyl (2S)-2-amino-3-(4-hydroxy-3,5-diiodo-Phenyl)propanoate;hydrochloride | -5.771 | -37.981 | 1 | Gln^219^ |
|  | 46737868 | N-(3-Hydroxy-2-iodo-4-pyridyl)acetamide | -5.315 | -33.317 | 1 | Gln^219^ |
|  | 19785608 | N-(2-Fluoro-4-iodo-phenyl)methanesulfonamide | -4.939 | -35.947 | - | - |
|  | 15322 | 1-Iodo-4-phenyl-benzene | -4.784 | -34.283 | - | - |
|  | 142891 | Ethyl 4-iodobenzoate | -4.78 | -36.754 | 1 | Thr^257^ |
|  | 46737871 | N-(5-Iodo-4-oxo-1H-pyridin-3-yl)acetamide | -4.778 | -34.897 | 1 | Asp^277^, |
|  | 4907056 | 4-Iodo-3,5-dimethyl-aniline | -4.773 | -25.955 | - | - |
|  | 2737343 | 2-Fluoro-4-iodo-1-(trifluoromethyl)benzene | -4.767 | -22.516 | - | - |
|  | 49761645 | 1-(Benzenesulfonyl)-2,3-diiodo-5-methyl-pyrrolo[2,3-b]pyridine | -4.763 | -45.833 | 1 | Thr^285^ |
|  | 11301181 | Benzyl N-(2-iodoethyl)carbamate | -4.749 | -36.331 | 1 | Gln^219^ |
|  | 689360 | ethyl 2-(4-iodoanilino)acetate | -4.73 | -35.72 | 1 | Thr^285^ |
|  | 50989463 | 6-Iodo-3,4-dihydro-2H-pyrano[3,2-b]pyridine | -4.722 | -29.242 | 1 | Gln^219^ |
|  | 2773407 | 1-(Ethoxymethyl)-2-iodo-imidazole | -4.716 | -24.138 | 1 | Gln^219^ |
|  | 7009501 | 2-Fluoro-3-iodo-pyridine | -4.701 | -21.352 | 1 | Gln^219^ |
|  | 46736822 | 3-(Dimethoxymethyl)-4-iodo-5-methoxy-pyridine | -4.686 | -32.329 | - | - |
|  | 46318220 | 6-Iodo-2,3-dihydro-1H-pyrido[2,3-b][1,4]oxazine | -4.684 | -30.047 | 1 | Gln^219^ |
|  | 76348 | 1-Iodo-4-(4-iodophenyl)benzene | -4.673 | -36.191 | - | - |
|  | 45933690 | 4-Iodo-3,5-bis(trifluoromethyl)-1H-pyrazole | -4.696 | -30.041 | - | - |
|  | 88542 | 1-Iodo-3-phenyl-benzene | -4.658 | -29.42 | - | - |
|  | 252610 | 3-Iodobenzaldehyde | -4.644 | -24.052 | 1 | Gln^219^ |
|  | 74373 | 4-Iodobenzoyl chloride | -4.642 | -27.087 | 1 | Gln^219^ |
|  | 879933 | 4-Iodo-N,N-dimethyl-benzamide | -4.635 | -32.817 | 1 | Gln^219^ |
|  | 10998636 | 3-Fluoro-4-iodo-pyridine | -4.632 | -21.548 | 1 | Gln^219^ |
|  | 2774516 | 2-Fluoro-4-iodo-benzoic acid | -4.629 | -27.8 | 1 | Gln^219^ |
|  | 49761550 | 6,8-Diiodo-3,4-dihydro-2H-pyrano[3,2-b]pyridine | -4.606 | -34.849 | 1 | Gln^219^ |
|  | 522722 | 2-Fluoro-6-iodo-benzonitrile | -4.585 | -26.646 | 1 | Gln^219^ |
|  | 22366768 | 3-Fluoro-4-iodo-benzoic acid | -4.585 | -28.231 | 1 | Gln^219^ |
|  | 13158583 | 5-Fluoro-2-iodo-benzoic acid | -4.585 | -27.174 | 1 | Thr^257^ |
|  | 11959096 | 5-Iodo-1H-pyrimidin-2-one | -4.615 | -27.389 | 1 | Gln^219^ |
|  | 615644 | 2-Iodo-N,N-dimethyl-benzamide | -4.567 | -35.721 | 1 | Thr^257^ |
|  | 2778928 | 2-Fluoro-5-iodo-benzonitrile | -4.544 | -25.05 | 1 | Thr^257^ |
|  | 11776298 | 2-Fluoro-4-iodo-3-methyl-pyridine | -4.541 | -21.391 | - | - |
|  | 2773390 | 4-Iodo-N,N-dimethyl-imidazole-1-sulfonamide | -4.537 | -33.634 | 1 | Gln^219^ |
|  | 12759111 | 2,3-Diiodopyridine | -4.534 | -24.717 | 1 | Gln^219^ |
|  | 458353 | 4-Iododibenzothiophene | -4.524 | -35.406 | - | - |
|  | 2778925 | 2-Fluoro-5-iodo-benzoic acid | -4.522 | -28.399 | 1 | Gln^219^ |
|  | 7059641 | (2R)-2-Amino-3-(4-hydroxy-3,5-diiodo-phenyl)propanoic acid | -4.983 | -30.59 | 1 | Gln^219^ |
|  | 67993 | 1-Iodo-4-(trifluoromethyl)benzene | -4.496 | -21.678 | - | - |
|  | 46911832 | 2-Fluoro-5-iodo-phenol | -4.568 | -26.549 | 2 | Gln^219^  Ser^260^ |
|  | 46737874 | 3-(Dimethoxymethyl)-5-iodo-pyridine | -4.458 | -27.667 | - | - |
|  | 15750131 | 6-Iodo-N,N-dimethyl-pyrazin-2-amine | -4.452 | -26.636 | 1 | Gln^219^ |
|  | 875746 | N-ethyl-4-iodo-benzamide | -4.443 | -32.939 | 1 | Asp^277^, |
|  | 16495276 | Ethyl 3-amino-3-(4-iodophenyl)propanoate;hydrochloride | -6.214 | -34.771 | 1 | Gln^219^ |
|  | 3681971 | Ethyl 6-iodoimidazo[1,2-a]pyridine-2-carboxylate | -4.991 | -33.541 | 2 | Gln^219^  Asn^264^ |
|  | 89957 | 2-Iodo-6-methyl-pyridin-3-ol | -4.931 | -26.889 | 1 | Gln^219^ |
|  | 53399604 | 1-Fluoro-3-iodo-5-methoxy-benzene | -4.414 | -24.174 | 1 | Gln^219^ |
|  | 49761552 | 6-Iodo-2,3-dihydro-[1,4]dioxino[2,3-b]pyridine | -4.414 | -28.832 | 1 | Gln^219^ |
|  | 24728217 | 6-Fluoro-3-iodo-2H-indazole | -4.42 | -28.368 | 1 | Ser^260^ |
|  | 46736796 | 2,5-Diiodo-3-methoxy-pyridine | -4.41 | -28.734 | 1 | Gln^219^ |
|  | 2773386 | 4,5-Diiodo-N,N-dimethyl-imidazole-1-sulfonamide | -4.407 | -36.991 | - | - |
|  | 643439 | 2-Iodobenzaldehyde | -4.4 | -24.817 | 1 | Gln^219^ |
|  | 46318097 | 2-Fluoro-3-iodo-6-pyrrolidin-1-yl-pyridine | -4.383 | -34.267 | - | - |
|  | 459500 | 5-Iodo-1H-pyridin-2-one | -4.38 | -28.454 | 1 | Gln^219^ |
|  | 45361774 | (5-Fluoro-4-iodo-pyrrolo[2,3-b]pyridin-1-yl)-triisopropyl-silane | -4.374 | -39.707 | - | - |
|  | 185694 | 2-Fluoro-4-iodo-aniline | -4.349 | -22.758 | - | - |
|  | 18454778 | 3-Fluoro-5-iodo-pyridine | -4.346 | -21.46 | 1 | Gln^219^ |
|  | 10361 | 4-Fluoro-3-iodo-benzoic acid | -4.343 | -28.519 | 1 | Gln^219^ |
|  | 76467 | 4-Iodobenzonitrile | -4.341 | -25.707 | 1 | Thr^257^ |
|  | 131506 | 5-Iodoindan-2-amine | -4.339 | -25.63 | - | - |
|  | 60146002 | 5-Iodoindan-2-amine;hydrochloride | -4.339 | -25.63 | - | - |
|  | 2758988 | 3-Fluoro-5-iodo-benzonitrile | -4.329 | -28.725 | 1 | Gln^219^ |
|  | 2759358 | 2-Iodobenzonitrile | -4.319 | -25.035 | 1 | Gln^219^ |
|  | 46736749 | 2,6-Diiodo-3,5-dimethoxy-pyridine | -4.318 | -33.594 | - | - |
|  | 2782196 | N-(2-Fluoro-4-iodo-phenyl)acetamide | -4.312 | -29.961 | 1 | Gln^219^ |
|  | 44754791 | Methyl 2-fluoro-4-iodo-pyridine-3-carboxylate | -4.297 | -28.288 | 1 | Gln^219^ |
|  | 46737827 | 5-Fluoro-3-iodo-1H-pyridin-2-one | -4.291 | -27.415 | 1 | Gln^219^ |
|  | 69676 | 1-Iodo-4-methoxy-benzene | -4.279 | -22.062 | 1 | Gln^219^ |
|  | 2783179 | 2-Fluoro-5-iodo-pyridine | -4.261 | -22.105 | 1 | Gln^219^ |
|  | 46318169 | 2-Fluoro-3,4-diiodo-6-pyrrolidin-1-yl-pyridine | -4.255 | -35.635 | - | - |
|  | 335838 | 2,5-Diiodo-1-methyl-imidazole | -4.219 | -24.065 | 1 | Gln^219^ |
|  | 20029093 | 3-Fluoro-2-iodo-aniline | -4.215 | -23.908 | - | - |
|  | 98577 | 3,5-Diiodobenzoic acid | -4.184 | -31.103 | 1 | Gln^219^ |
|  | 23435549 | 2,5-Diiodopyridin-3-ol | -4.885 | -30.662 | 1 | Gln^219^ |
|  | 46736877 | N-(3-Hydroxy-2-iodo-4-pyridyl)-2,2-dimethyl-propanamide | -4.653 | -37.263 | 2 | Leu^218^, Gln^219^ |
|  | 12271 | 3-Iodoaniline | -4.166 | -24.494 | 1 | Asp^277^, |
|  | 75361260 | Ethyl 3,5-dichloro-2-iodo-benzoate | -4.159 | -38.104 | - | - |
|  | 20058425 | 1-ethyl-4-iodo-pyrazole | -4.152 | -22.576 | 1 | Gln^219^ |
|  | 49761561 | 7-Iodo-3,5-dihydro-2H-[1,4]dioxino[2,3-b]pyridin-8-one | -4.211 | -28.132 | 1 | Gln^219^ |
|  | 332387 | 4,5-diiodo-1-methyl-imidazole | -4.133 | -24.417 | 1 | Gln^219^ |
|  | 49761561 | 7-Iodo-3,5-dihydro-2H-[1,4]dioxino[2,3-b]pyridin-8-one | -5.496 | -30.475 | 1 | Gln^219^ |
|  | 17779156 | 1-(2-Iodoethyl)pyrrolidin-2-one | -4.111 | -25.813 | 1 | Gln^219^ |
|  | 2778247 | 2-Iodobenzenesulfonic acid | -4.111 | -25.193 | 1 | Gln^219^ |
|  | 50989285 | 4-Iodo-2,3-dimethoxy-pyridine | -4.107 | -27.632 | - | - |
|  | 263416 | 5-Iodo-2,4-dimethoxy-pyrimidine | -4.103 | -26.707 | 1 | Gln^219^ |
|  | 19597673 | 6-Fluoro-2-iodo-pyridin-3-ol | -4.876 | -26.989 | 1 | Gln^219^ |
|  | 15760295 | 4,6-Diiodopyrimidine | -4.083 | -23.59 | 1 | Gln^219^ |
|  | 11739913 | 2,6-Diiodo-3-methoxy-pyridine | -4.068 | -28.996 | - | - |
|  | 1519483 | 3-Fluoro-2-iodo-pyridine | -4.067 | -21.832 | 1 | Gln^219^ |
|  | 2783156 | 2-Fluoro-6-iodo-aniline | -4.065 | -22.323 | - | - |
|  | 19617512 | 2-(4-Iodo-3,5-dimethyl-pyrazol-1-yl)acetic acid | -4.053 | -25.487 | 1 | Gln^219^ |
|  | 18455991 | N-(5-Fluoro-3-iodo-2-pyridyl)-2,2-dimethyl-propanamide | -4.039 | -37.012 | - | - |
|  | 627400 | 2-Iodobenzenesulfonyl chloride | -4.033 | -33.142 | - | - |
|  | 123492 | 2-Fluoro-4-iodo-1-methyl-benzene | -3.981 | -22.556 | - | - |
|  | 2784731 | 2,6-Diiodopyridin-3-ol | -4.67 | -30.413 | 1 | Gln^219^ |
|  | 967640 | N-Ethyl-2-iodo-benzamide | -3.971 | -37.969 | 1 | Leu^218^, |
|  | 11002078 | 2,6-Diiodopyrazine | -3.967 | -22.914 | 1 | Gln^219^ |
|  | 11995 | 2-Iodoaniline | -3.967 | -23.132 | - | - |
|  | 14006092 | Ethyl 2-chloro-6-hydroxy-benzoate | -4.346 | -40.959 | 1 | Gln^219^ |
|  | 131506 | 5-Iodoindan-2-amine | -3.938 | -19.737 | 1 | Asp^277^, |
|  | 60146002 | 5-Iodoindan-2-amine;hydrochloride | -3.938 | -19.737 | 1 | Asp^277^, |
|  | 2773382 | 4,5-Diiodo-1H-imidazole | -3.932 | -21.437 | 2 | Gln^219^  Asp^277^, |
|  | 2774525 | 4-Fluoro-1-iodo-2-methyl-benzene | -3.921 | -22.434 | - | - |
|  | 3409621 | N-Ethyl-3-iodo-benzamide | -3.917 | -34.796 | 1 | Gln^219^ |
|  | 19261611 | 2-Fluoro-4-iodo-1-nitro-benzene | -3.9 | -29.502 | 1 | Gln^219^ |
|  | 10893 | 4-Iodoaniline | -3.864 | -22.746 | - | - |
|  | 259086 | 1-Fluoro-3-iodo-5-nitro-benzene | -3.853 | -30.826 | 1 | Gln^219^ |
|  | 46737868 | N-(3-Hydroxy-2-iodo-4-pyridyl)acetamide | -4.783 | -32.009 | 2 | Gln^219^ |
|  | 45361762 | 2,6-Diiodo-5-methoxy-pyridin-3-ol | -4.188 | -29.647 | 1 | Gln^219^ |
|  | 2774523 | 1-Fluoro-3-iodo-2-methyl-benzene | -3.817 | -22.299 | - | - |
|  | 827981 | 2-Iodo-1,3-benzothiazole | -3.813 | -29.1 | 1 | Gln^219^ |
|  | 45933690 | 4-Iodo-3,5-bis(trifluoromethyl)-1H-pyrazole | -5.471 | -31.676 | - | - |
|  | 12065 | 4-Hydroxy-3,5-diiodo-benzoic acid | -4.049 | -31.227 | 1 | Thr^257^ |
|  | 140924 | 1-Iodo-3,5-dimethyl-benzene | -3.795 | -22.409 | - | - |
|  | 639791 | Ethyl (E)-3-iodoprop-2-enoate | -3.767 | -24.162 | 1 | Gln^219^ |
|  | 16638330 | 4-Iodo-1H-pyridazin-6-one | -4.002 | -27.425 | 2 | Gln^219^  Asn^264^ |
|  | 3836324 | 1-Fluoro-2-iodo-3-methyl-benzene | -3.753 | -19.637 | - | - |
|  | 50998944 | 1-Ethyl-3-iodo-pyrazole | -3.752 | -22.934 | - | - |
|  | 70731 | 2-Iodo-1,4-dimethyl-benzene | -3.738 | -23.493 | - | - |
|  | 630554 | 4,5-Diiodo-2-methyl-1H-imidazole | -3.736 | -25.415 | - | - |
|  | 954258 | 1-(Bromomethyl)-4-iodo-benzene | -3.726 | -27.456 | - | - |
|  | 46737868 | N-(3-Hydroxy-2-iodo-4-pyridyl)acetamide | -4.317 | -31.453 | 2 | Gln^219^ |
|  | 85111 | 2-Quinolylhydrazine | -4.832 | -31.268 | 2 | Gln^219^  Thr^257^ |
|  | 16495276 | Ethyl 3-amino-3-(4-iodophenyl)propanoate;hydrochloride | -5.439 | -35.536 | 1 | Gln^219^ |
|  | 56777166 | Methyl (2S)-2-amino-3-(4-hydroxy-3,5-diiodo-Phenyl)propanoate;hydrochloride | -4.659 | -37.12 | 1 | Gln^219^ |
|  | 69097 | 2-Iodo-1,3-dimethyl-benzene | -3.662 | -20.874 | - | - |
|  | 252612 | 2-Hydroxy-5-iodo-benzaldehyde | -4.625 | -27.361 | 1 | Gln^219^ |
|  | 2783155 | 1-Fluoro-3-iodo-2-nitro-benzene | -3.654 | -29.259 | 1 | Gln^219^ |
|  | 12065 | 4-Hydroxy-3,5-diiodo-benzoic acid | -4.274 | -30.699 | 1 | Thr^257^ |
|  | 2758568 | Dimethyl 5-iodobenzene-1,3-dicarboxylate | -3.642 | -43.68 | 1 | Thr^257^ |
|  | 45480261 | 5-Iodo-2-oxo-1H-pyridine-3-carbonitrile | -4.109 | -28.276 | 1 | Thr^257^ |
|  | 19361948 | 4-Fluoro-2-iodo-1-nitro-benzene | -3.581 | -30.468 | - | - |
|  | 12270 | 1,3-Diiodobenzene | -3.526 | -23.401 | - | - |
|  | 7023570 | 2-Fluoro-4-iodo-pyridine | -3.523 | -20.878 | 1 | Gln^219^ |
|  | 45480261 | 5-Iodo-2-oxo-1H-pyridine-3-carbonitrile | -3.819 | -35.396 | 1 | Thr^257^ |
|  | 12208 | 1,4-Diiodobenzene | -3.48 | -24.456 | - | - |
|  | 50989461 | 6-Iodo-3,4-dihydro-2H-pyrano[3,2-b]pyridine-8-carbaldehyde | -3.477 | -32.993 | 1 | Ser^260^ |
|  | 15561305 | 4,6-Diiodo-2-methyl-pyrimidine | -3.465 | -26.166 | 1 | Thr^285^ |
|  | 16638330 | 4-Iodo-1H-pyridazin-6-one | -4.088 | -28.901 | 2 | Gln^219^  Asp^277^, |
|  | 56777166 | Methyl (2S)-2-amino-3-(4-hydroxy-3,5-diiodo-Phenyl)propanoate;hydrochloride | -4.701 | -36.991 | 1 | Gln^219^ |
|  | 70725 | 1-Fluoro-3-iodo-benzene | -3.393 | -19.994 | - | - |
|  | 10911297 | Ethyl 3-iodopropanoate | -3.387 | -23.136 | 1 | Thr^285^ |
|  | 71832992 | 1-Ethyl-5-iodo-pyrazole | -3.373 | -22.126 | 1 | Gln^219^ |
|  | 322557 | 2,4-Diiodo-5-methyl-1H-imidazole | -4.093 | -24.351 | 2 | Gln^219^  Asp^277^, |
|  | 19597673 | 6-Fluoro-2-iodo-pyridin-3-ol | -3.519 | -20.227 | 1 | Gln^219^ |
|  | 46736877 | N-(3-Hydroxy-2-iodo-4-pyridyl)-2,2-dimethyl-propanamide | -4.332 | -34.289 | 1 | Asp^277^, |
|  | 11221242 | 2,4-Diiodopyrimidine | -3.246 | -24.273 | 1 | Thr^285^ |
|  | 22832107 | 5-Fluoro-2-iodo-pyridine | -3.231 | -19.869 | 1 | Thr^285^ |
|  | 12354 | 1,5-Diiodopentane | -3.166 | -23.841 | 1 | Gln^219^ |
|  | 2784731 | 2,6-Diiodopyridin-3-ol | -3.473 | -24.376 | 1 | Gln^219^ |
|  | 19785608 | N-(2-Fluoro-4-iodo-phenyl)methanesulfonamide | -3.996 | -31.617 | - | - |
|  | 322557 | 2,4-Diiodo-5-methyl-1H-imidazole | -3.293 | -23.946 | - | - |
|  | 11427514 | 2,6-Diiodopyridine | -3.047 | -21.047 | 1 | Thr^285^ |
|  | 14006092 | Ethyl 2-chloro-6-hydroxy-benzoate | -3.465 | -31.959 | 2 | Gln^219^  Ser^260^ |
|  | 46315209 | 3-Iodo-5-(trifluoromethyl)-1H-pyridin-2-one | -4.907 | -25.816 | 1 | Gln^219^ |
|  | 7059641 | (2R)-2-Amino-3-(4-hydroxy-3,5-diiodo-phenyl)propanoic acid | -3.368 | -35.254 | 2 | Gln^219^  Ser^260^ |
|  | 11575 | Iodobenzene | -2.994 | -20.412 | - | - |
|  | 45361762 | 2,6-Diiodo-5-methoxy-pyridin-3-ol | -4.789 | -33.415 | 1 | Gln^219^ |
|  | 613883 | 4-Iodo-3,5-dimethyl-isoxazole | -2.795 | -20.36 | - | - |
|  | 16217505 | (4-Iodophenyl)methanamine;hydrochloride | -4.727 | -22.579 | 1 | Gln^219^ |
|  | 49761651 | 1-(6-Iodo-2,3-dihydropyrido[2,3-b][1,4]oxazin-1-yl)-2,2-dimethyl-propan-1-one | -2.71 | -32.945 | 1 | Gln^219^ |
|  | 23435549 | 2,5-Diiodopyridin-3-ol | -3.199 | -23.183 | 1 | Gln^219^ |
|  | 46911832 | 2-Fluoro-5-iodo-phenol | -3.755 | -21.901 | 1 | Gln^219^ |
|  | 11959096 | 5-Iodo-1H-pyrimidin-2-one | -4.234 | -24.103 | 1 | Gln^219^ |
|  | 89957 | 2-Iodo-6-methyl-pyridin-3-ol | -2.986 | -21.583 | 2 | Gln^219^  Asp^277^, |
|  | 45361746 | 6-Iodo-2,3-dimethoxy-pyridine | -2.55 | -28.744 | 1 | Gln^219^ |
|  | 638126 | (E)-3-Iodoprop-2-enoic acid | -2.491 | -16.258 | - | - |
|  | 3833565 | 1,1-Difluoro-2-iodo-ethane | -2.458 | -14.727 | 2 | Gln^219^  Thr^257^ |
|  | 736854 | 3-Hydroxy-4-iodo-benzoic acid | -4.458 | -26.064 | 2 | Gln^219^  Thr^257^ |
|  | 46736769 | 5-Iodo-2,3-dimethoxy-pyridine | -2.437 | -28.546 | 1 | Gln^219^ |
|  | 15475546 | 3-Fluoro-4-iodo-aniline | -2.429 | -25.808 | - | - |
|  | 16217505 | (4-Iodophenyl)methanamine;hydrochloride | -2.427 | -25.753 | 2 | Leu^218^, Gln^219^ |
|  | 2784731 | 2,6-Diiodopyridin-3-ol | -3.562 | -23.624 | 1 | Gln^219^ |
|  | 24728217 | 6-Fluoro-3-iodo-2H-indazole | -4.71 | -29.92 | 2 | Gln^219^  Asp^277^, |
|  | 6346 | Diiodomethane | -2.114 | -18.365 | - | - |
|  | 46318064 | 8-Iodo-3,4-dihydro-2H-pyrano[3,2-c]pyridine | -3.373 | -27.763 | - | - |
|  | 46736877 | N-(3-Hydroxy-2-iodo-4-pyridyl)-2,2-dimethyl-propanamide | -2.482 | -28.247 | - | - |
|  | 69804 | 1-Fluoro-2-iodo-ethane | -1.848 | -12.856 | - | - |
|  | 19597673 | 6-Fluoro-2-iodo-pyridin-3-ol | -3.619 | -20.178 | 1 | Gln^219^ |
|  | 46737827 | 5-Fluoro-3-iodo-1H-pyridin-2-one | -4.191 | -25.726 | 1 | Gln^219^ |
|  | 89957 | 2-Iodo-6-methyl-pyridin-3-ol | -3.006 | -17.894 | 1 | Thr^285^ |
|  | 45361762 | 2,6-Diiodo-5-methoxy-pyridin-3-ol | -0.97 | -28.987 | - | - |
|  | 23435549 | 2,5-Diiodopyridin-3-ol | -0.462 | -22.92 | 1 | Gln^219^ |

**Supplementary Table 2.** Lethal and sub-lethal concentrations of tested compounds against PWN.

| **Si No** | **Compounds** | **LC_50_** | **LC_90_** | **Mechanism** | **Reference** |
| --- | --- | --- | --- | --- | --- |
| 1 | 5F4IPP | <13.1 µg/mL | 26.2 µg/mL | GluCl interaction | This study |
| 2 | 6-Iodoindoline | 12.25 µg/mL | 24.3 µg/mL | - | This study |
| 3 | 7-Fluoro-5-iodoindole | 13.05 µg/mL | 26.1 µg/mL | - | This study |
| 4 | 5-Iodoindole | 12.15 µg/mL | 24.3 µg/mL | Methuosis | ^14^ |
| 5 | Indole | 175.5 µg/mL | 234 µg/mL | - | ^14^ |
| 6 | Abamectin | 5 µg/mL | 20 µg/mL | GluCl activation | ^14^ |

**Supplementary Table 3.** Toxicity of 5-iodoindole and indole on male BALB-c mice. Four mice were tested per group.

|  | **Treatment** | **Mice survival (%)** |
| --- | --- | --- |
| Control group | DMSO (vehicle) | 100 |
| 5-Iodoindole | LC_50_:11,040 mg/kg  LC_75_: 12,000 mg/kg  LC_100_: 14,400 mg/kg | 50  25  0 |
| Indole | LC_50_: 960 mg/kg  LC_75_: 1,920 mg/kg  LC_100_: 2,400 mg/kg | 50  25  0 |

*
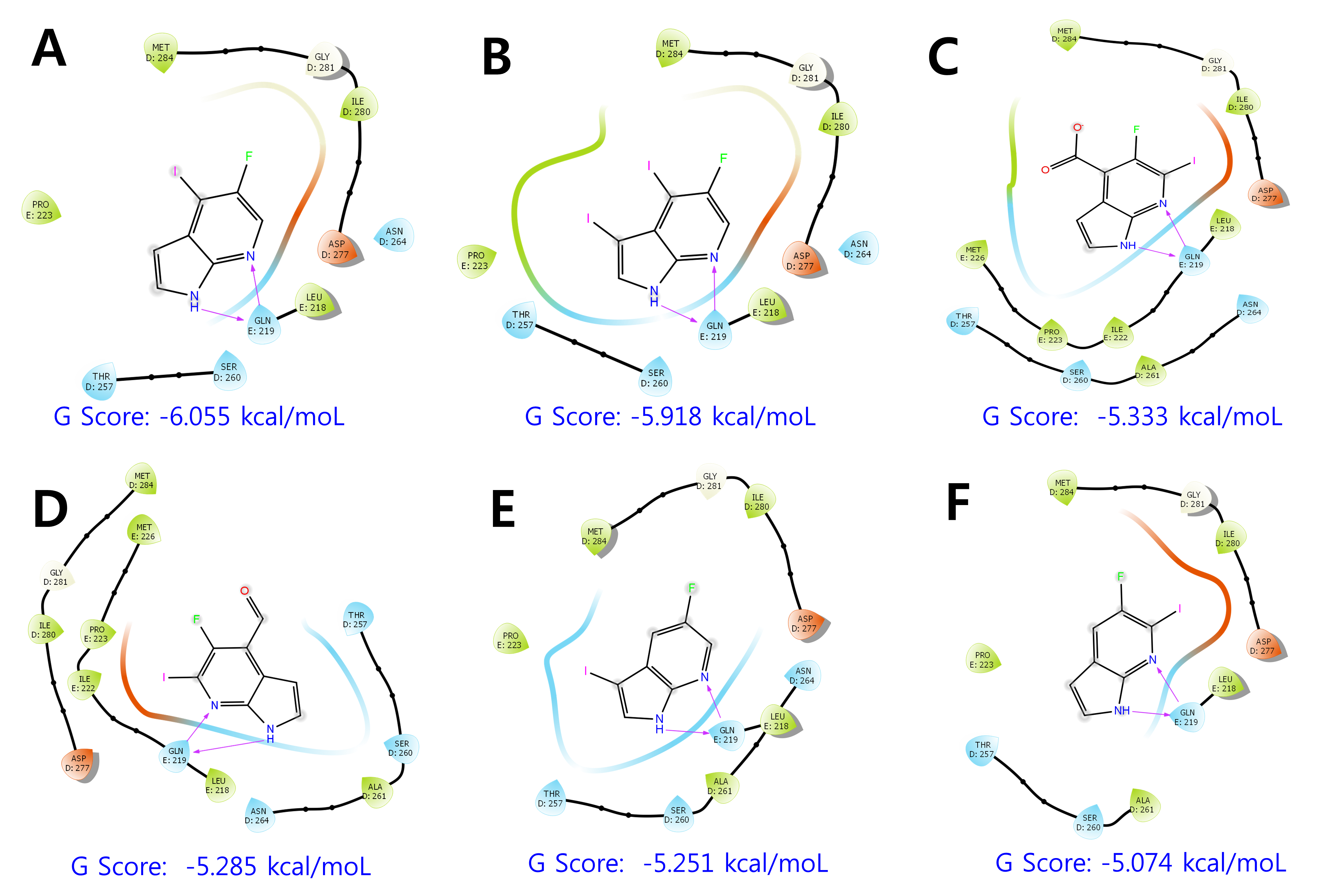
***Supplementary Figure S1.** Interactions of 1H-pyrrolo [2, 3-b] pyridine (7-azaindole) derivatives with GluCl receptor. (A) 5F4IPP, (B) 5-fluoro-3,4-diiodo-1H-pyrrolo[2,3-b]pyridine, (C) 5-fluoro-6-iodo-1H-pyrrolo[2,3-b]pyridine-4-carboxylic acid, (D) 5-fluoro-6-iodo-1H-pyrrolo[2,3-b]pyridine-4-carbaldehyde, (E) 5-fluoro-3-iodo-1H-pyrrolo[2,3-b]pyridine, and (F) 5-fluoro-6-iodo-1H-pyrrolo[2,3-b]pyridine.

**.
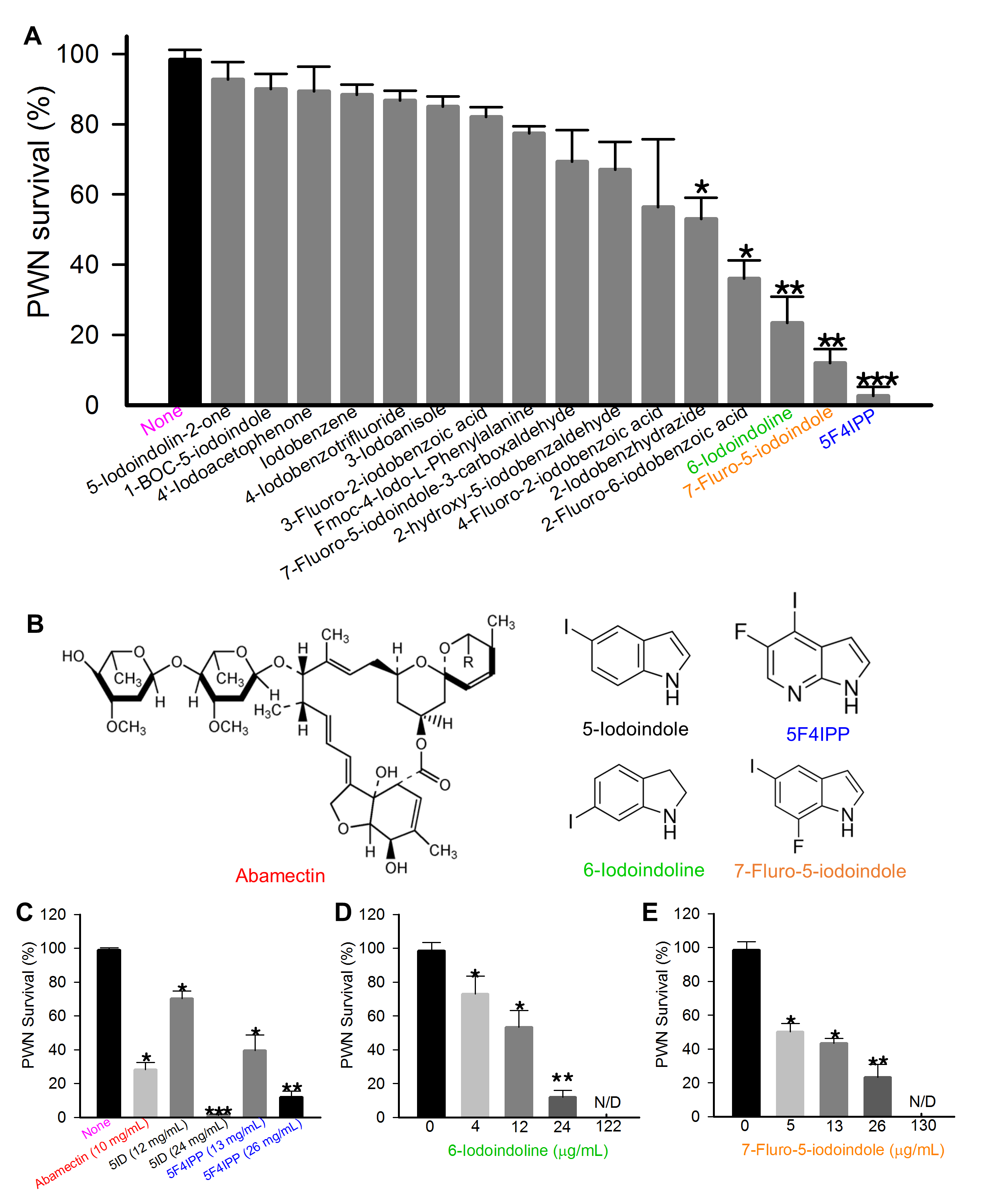
**

**Supplementary Figure S2.** (A) Nematicidal activities of indole based iodine-fluorine compounds (0.1 mM) against mixed stages of pinewood nematode (PWN; *B. xylophilus*). The graph shows the means ± SEMs of three measurements. * *P*<0.05, ** *P*<0.01, and *** *P*<0.001 vs. the non-treated controls. (B) Molecular structures of abamectin, 5F4IPP, 6-iodoindoline, 5-iodoindole, and 7-fluoro-5-iodoindole, (C) Comparative analysis of the lead compounds tested, (D) effect of 7-fluoro-5-iodoindole on instar stages of *B. xylophilus*, and (E) Effect of 7-fluoro-5-iodoindole on instar stages of *B. xylophilus.* The graphs show the means ± SEMs of three repetitions. * *P*<0.05, ** *P*<0.01, and *** *P*<0.001 vs. the non-treated controls.

**Supplementary Figure S3.** Time-lapse images of the locomotor behavior of *B. xylophilus*. Time-lapse images showing the sinusoidal and thrashing movements of J2s in sterile distilled water. (A) Control, (B) 5F4IPP (13 µg/mL), and (C) 5F4IPP (26 µg/mL). Scale bars = 20 µm.

**Supplementary Figure S4.** Time-lapse images of the sequential hatching process of *B. xylophilus* J2 eggs in distilled water. The arrowhead indicates the egg shell. Scale bars = 50 µm.

**
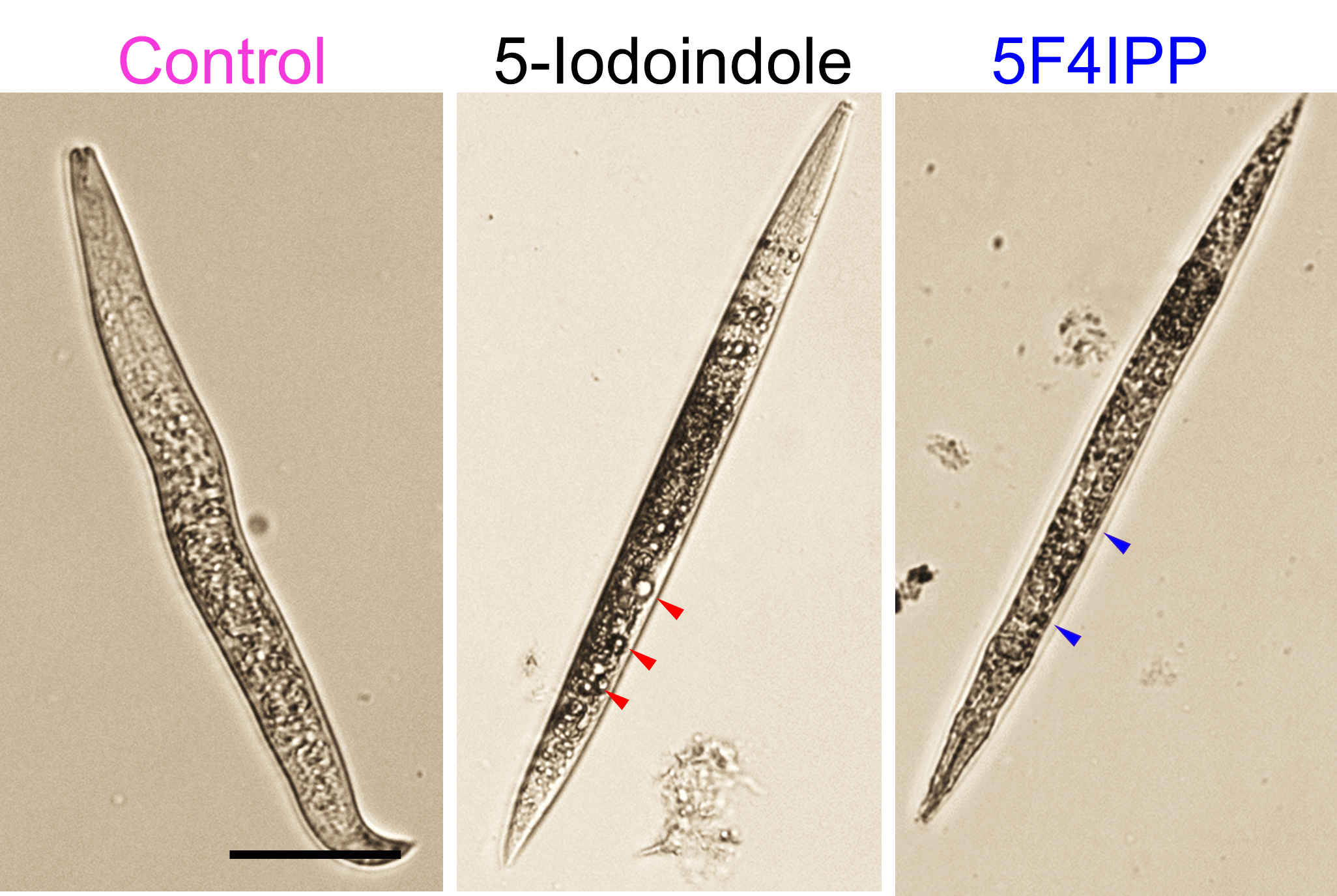
**

**Supplementary Figure S5.** Effects of 5-iodoindole and 5F4IPP on the J3 developmental stage of *M. incognita*. Red arrowhead indicates vacuoles and the blue arrowhead indicate membrane shrinkage. Scale bars = 50 µm.

**
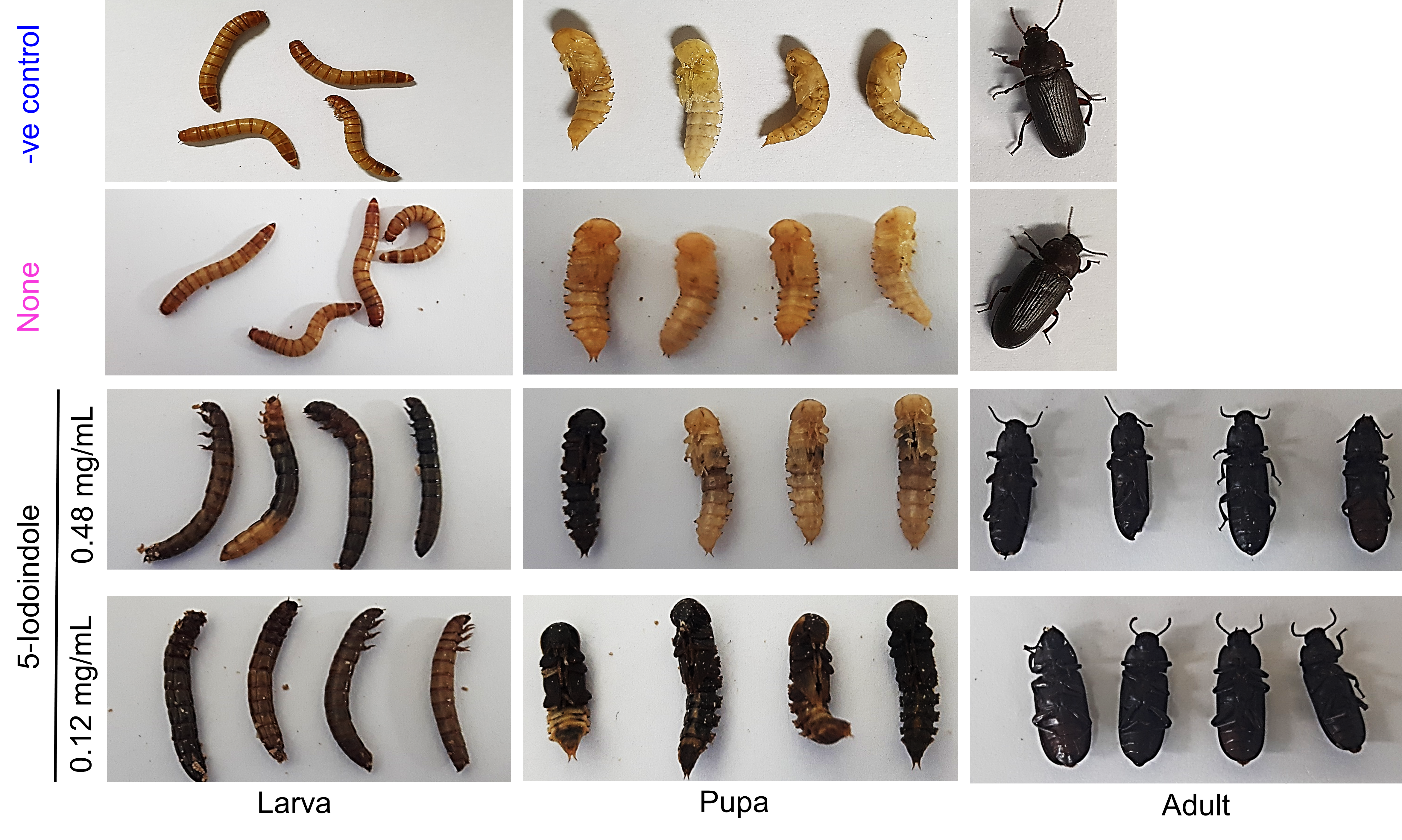
**

**Supplementary Figure S6.** Effect of 5-iodoindole on instar stages of the model insect, *T. molitor.* Sequential necrotic events in larvae, pupae and adults after an intradermal injection of 5-iodoindole. 0.1% DMSO was used as a negative control.

**Supplementary Figure S7.** Effect of methylindole derivatives (0.1 mM) on *B. xylophilus* mixed developmental stages. Blue colors represent 1-methyl indoles derivatives. The graph shows the means ± SEMs of three repetitions. ** *P*<0.01 vs. the non-treated controls.
